# A cross-sectional latent class analysis of depressive and anxiety symptoms in people aged 65 and older: Identifying subgroups and related factors

**DOI:** 10.1097/MD.0000000000050328

**Published:** 2026-08-21

**Authors:** Jing Zhang, Feng Li, YunTong Yao, YuQing Huang, YiXia Zhang, YangWen Yu, Xu Luo

**Affiliations:** a Guizhou Provincial Center for Disease Control and Prevention, Guiyang, China; b School of Nursing, Zunyi Medical University, Zunyi, China; c School of Medical Information Engineering, Zunyi Medical University, Zunyi, China.

**Keywords:** aging, anxiety, chronic disease, depression, latent class analysis, latent class model

## Abstract

As the older population grows, depression and anxiety have become common mental health problems that significantly undermine the quality of life of older persons. Improving older people’s mental health is vital for their quality of life. Before developing targeted interventions, priority must be given to identifying key factors affecting mental health to address the significant individual differences within this population. The aim was to identify subgroups of depressive/anxiety symptoms in the older population and factors influencing their mental health. Secondary analysis of mental health data of elderly people aged 65 years and older in Guizhou Province, China. Latent Class Modeling was developed using Mplus 8.3 software to examine the factors that influence the occurrence of anxiety and depression in different subgroups of older adults. Chi-square test and unordered multivariate logistic regression were used for detailed analysis from 3 perspectives (demographic characteristics, physical condition and family relationships, and lifestyle). A total of 4 potential category subgroups were identified in this study: the severe depressive and anxiety symptoms group (Class 1, N = 406, 1.46%), the moderate depressive and anxiety symptoms group (Class 2, N = 1834, 6.58%), the mild depressive and anxiety symptoms group (Class 3, N = 3789, 13.59%), and the group without depressive or anxiety symptoms (Class 4, N = 21,851, 78.38%). Significant individual differences in symptoms of depression and anxiety exist among people aged 65 and older. The results of the disordered multiple logistic regression analysis revealed that the following factors contribute to differences in depression and anxiety symptoms among older adults: gender, body mass index, ethnicity, occupation before retirement, marital status, physical disability status, independence in activities of daily living, number of chronic diseases, smoking and drinking habits, dietary habits, recreational activities, physical activity, and sleep quality. In the context of global aging, the mental health of older adults is paramount. Given significant heterogeneity in their mental health status, health and policy sectors should develop individualized interventions targeting specific subgroups and their risk factors to effectively reduce anxiety and depression.

## 1. Introduction

Global population aging, driven by increased life expectancy, declining fertility, and medical advancements, has become a significant social challenge worldwide.^[1]^ Data show that China has the largest population aged 60 and above globally, indicating a notable aging trend.^[2]^ Among the elderly population, chronic diseases have replaced infectious and neonatal diseases as the leading cause of death.^[3]^

Chronic diseases are typically irreversible and persistent. Multiple chronic conditions predispose older adults to common mental health issues like anxiety and depression. Among Chinese patients aged ≥65 years receiving care in primary care facilities, 30.6% exhibited depressive symptoms,^[4]^ and 21.1% exhibited anxiety symptoms.^[5]^ Depression and anxiety have been shown to adversely influence cognitive function and economic capacity in older adults, notably in those with amnestic mild cognitive impairment or vascular dementia.^[6,7]^ The worsening of depressive symptoms not only worsens general cognitive outcomes but also worsens economic capacity.^[8]^ In addition, severe anxiety and depressive symptoms in older adults significantly harm individual well-being and pose a public health concern. Poor mental health promotes health-risk behaviors, exacerbates chronic diseases, compromises social functioning, diminishes quality of life, and increases vulnerability to suicide.^[9]^ It also markedly increases healthcare utilization and expenditures, imposing a substantial burden on the healthcare system.^[10]^

Depressive and anxiety symptoms constitute the core features of depressive and anxiety disorders, respectively, and represent subclinical conditions associated with significant health impairment.^[11]^ Early psychological intervention represents a key strategy for preventing the onset of depression and anxiety disorders in older adults, thus potentially reducing disability rates. At present, the treatment of mental health issues in the elderly is divided into two principal categories: pharmacological and non-pharmacological.^[12]^ While medications, particularly antianxiety and antidepressant drugs, are commonly used,^[13,14]^ their potential risks and side effects: such as dependence, withdrawal, and increased fall risk in older adults: require careful consideration. Non-pharmacological approaches offer viable alternatives or complements to medication, helping mitigate adverse effects while achieving therapeutic outcomes. Understanding the factors that contribute to depressive and anxiety symptoms in older adults is essential for improving non-pharmacological interventions. Previous research has identified several risk factors for depression and anxiety in older adults, including female sex, low educational attainment, poor economic status, adverse family relationships, and the presence of chronic illness.^[4,5,15]^ Although the prevalence and risk factors of anxiety and depressive symptoms in older adults are well-documented, prior research often treats these symptoms as homogeneous constructs. This approach could obscure clinically distinct subgroups with unique symptom patterns, etiologies, and intervention needs, leaving the heterogeneity of mental health in this population inadequately addressed.

The latent class model, a statistical method used in psychology and sociology, identifies homogeneous and distinct subgroups within a sample based on observed behaviors, positing that latent categorical variables explain the relationships between observed variables.^[16–20]^ The model groups individuals with similar characteristics or behavioral patterns into classes, enhancing within-class homogeneity and between-class heterogeneity.^[21]^ The latent class analysis (LCA) aims to identify categories through categorical indicators, assuming that the underlying categorical variables explain the relationships between observed variables.^[22]^ Additionally, LCA surpasses the constraints of factor analysis and structural equation modeling that necessitate continuous latent variables.^[23]^ Zhou et al used LCA to divide women aged 65 and older into 3 mental health subgroups.^[24]^ This helped explain the heterogeneity among older women and elucidate unique aspects of their mental health. This approach identifies priority groups for psychological care, thereby supporting personalized interventions that cater to the diverse needs and preferences of older adults. This approach improves the efficiency of healthcare resource allocation, thus enabling the development of individualized, integrated, and recovery-oriented interventions.

This study examined older adults’ mental health in Guizhou Province (≥65 years) through 3 objectives: First, using LCA to identify classifications of depressive and anxiety symptoms; second, examining how demographic, physical, social, lifestyle, cognitive, and self-assessment factors influence these categories; finally, providing evidence for personalized interventions in resource-limited settings.

## 2. Methods

### 2.1. Participants

This study is a secondary analysis of the data. The study population comprised individuals aged 65 years and older, whose data were sourced from the electronic medical records system of the Guizhou Provincial Center for Disease Control and Prevention between January and December 2022. The cross-sectional survey was conducted using cluster sampling to assess the mental health of permanent residents aged 65 and above in 3 cities in Guizhou Province (Tongren, Guiyang, and Liupanshui). The survey aimed to preliminarily assess the mental health status of the elderly population in the province. First, Guiyang, Tongren, and Liupanshui were selected to represent the eastern, central, and western regions, respectively. Second, at least 2 subdistricts and 1 town were selected for survey locations in each city. However, during the survey, the Guiyang region covered 3 counties, the Liupanshui region covered 4 districts, and the Tongren region covered 1 county. This study adheres to the ethical principles of the Declaration of Helsinki and has been reviewed and approved by the Ethics Committee of the Affiliated Hospital of Zunyi Medical University (approval number KLLY-2024-234). Informed consent was obtained from all participants during the cross-sectional survey. Since this study uses a retrospective analysis of existing cross-sectional data, it is unnecessary to obtain informed consent from participants again who have already completed the survey.

**Figure 1.**
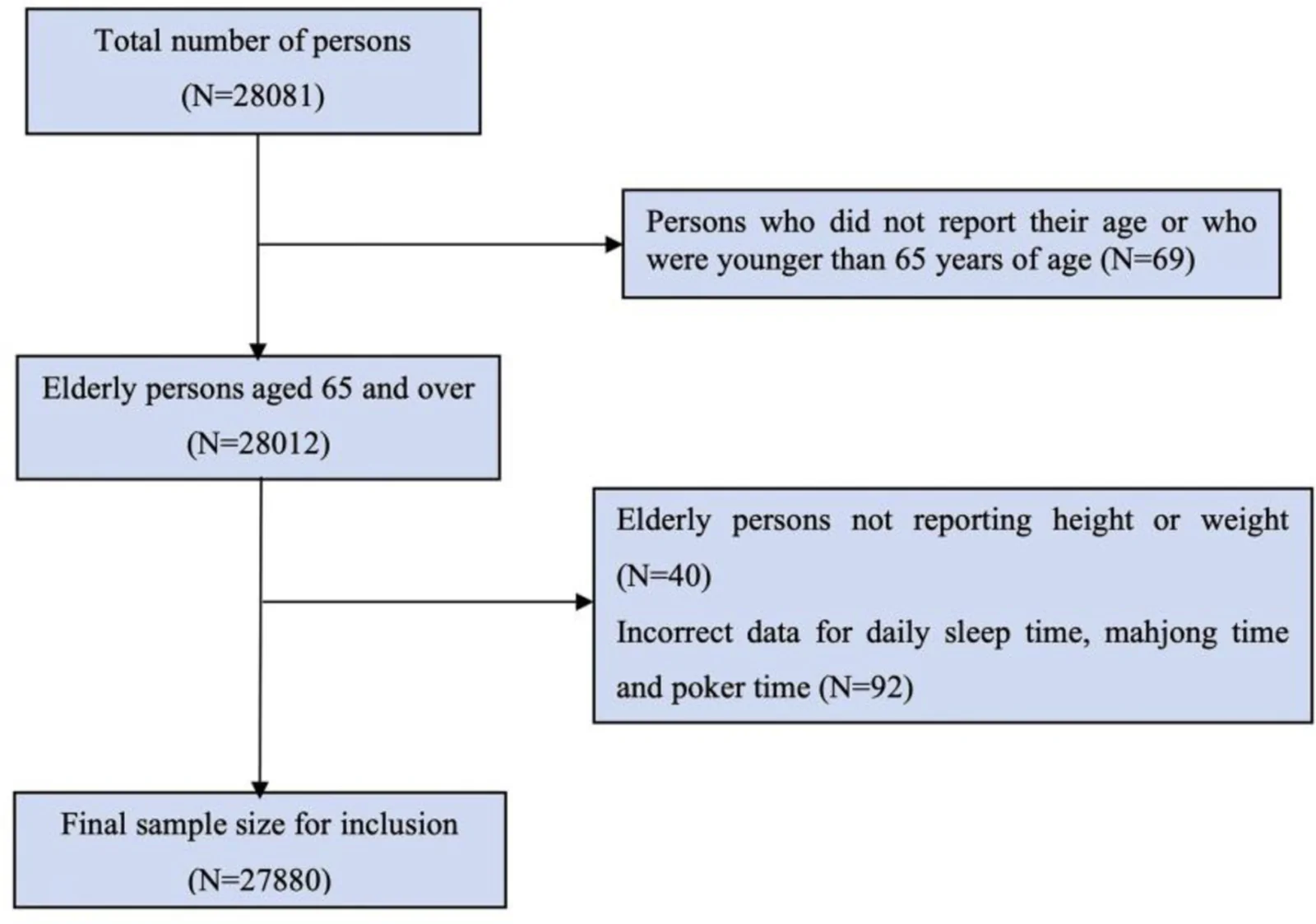
Screening flowchart for population inclusion.

A total of 28,081 older adults participated in this cross-sectional survey. After excluding 69 participants who did not report their age or who were younger than 65 years, 40 participants who did not report their height or weight, and 92 participants who misreported their data (daily sleep time, daily mahjong time, and daily poker time), 27,880 individuals remained for this final data analysis. For details on the data screening process, please refer to Figure 1.

### 2.2. Measures

Anxiety and depressive symptoms in older adults were assessed using the Generalized Anxiety Disorder Scale (GAD-7) and the Patient Health Questionnaire-9 (PHQ-9). The use of the PHQ-9 and GAD-7 in this study was dictated by their application in the original survey from which our data were derived. Notwithstanding the specific advantages of geriatric-specific instruments like the GDS-15 and GAI-SF, the PHQ-9 and GAD-7 are internationally recognized, brief screening tools with established validity and reliability in Chinese older adult populations.^[25,26]^ Their widespread use also facilitates comparisons with broader epidemiological research.

All items from the PHQ-9 and GAD-7, originally 4-category responses, were dichotomized to serve as indicators for a LCA of depressive and anxiety symptoms in older adults. See Supplementary Material 1, Supplemental Digital Content 1 for details of the scale.

#### 2.2.1. The Generalized Anxiety Disorder Scale

The GAD-7 scale^[27]^ assesses anxiety through 7 items with 4 responses: “not at all (0 points),” “several days (1 point),” “more than a week (2 points),” and “nearly every day (3 points).” Total scores range from 0 to 21; ≥5 indicates anxiety (mild: 5–9; moderate: 10–14; severe: 15–21). By the methodology of LCA, which is employed in treating categorical variables, the response “not at all” was assigned the value of “0.” The remaining responses were assigned the value of “1.” Its validity for older adults has been verified,^[25]^ and Cronbach’s α was 0.865 in this study.

#### 2.2.2. The PHQ-9

The PHQ-9 is a self-report scale comprising 9 questions about the frequency of depressive symptoms experienced by the study participant over the past 2 weeks. The PHQ-9, a depression screening tool^[28]^ assesses 9 symptoms across affective and somatic dimensions. Participants rate symptom frequency over 2 weeks using 4 options: not at all (0 points), several days (1 point), more than half the days (2 points), almost every day (3 points). Total scores range 0 to 27; ≥5 suggests depression (mild: 5 to 9; moderate: 10–14; severe: 15–27). Similarly, those indicating “several days,” “more than half of the days,” and “almost every day” were recoded as “1.” Validated for older adults,^[26]^ it shows good reliability (Cronbach’s alpha = 0.82) and validity (sensitivity = 0.97, specificity = 0.89) in rural populations. This study’s Cronbach’s α was 0.830.

#### 2.2.3. The ascertain dementia 8

The Ascertain Dementia 8 assesses cognitive function, with its Simplified Chinese version adapted from English and Traditional Chinese sources.^[29,30]^ Each question on the scale should be answered individually, with 1 point awarded for a positive response and no points for a negative response. A score of 0 to 1 indicates normal cognitive function, whereas a score of 2 or above suggests the possibility of cognitive impairment. Validated for detecting mild-to-severe cognitive impairment in older adults with memory complaints,^[31]^ it achieved Cronbach’s α = 0.911 in this study. The reliability test results for all scales are shown in Table 1.

**Table 1:** Scale reliability tests.

Scales	Cronbach Alpha	Item counts
GAD-7	0.865	7
PHQ-9	0.830	9
AD8	0.911	8

AD8 = Ascertain Dementia 8-item Interview, GAD-7 = Generalized Anxiety Disorder 7-item Scale, PHQ-9 = Patient Health Questionnaire-9.

### 2.3. Covariate

In this study, all of the variables were categorized into 3 groups: demographic characteristics, physical status and family relationships, and lifestyle.

The variables related to demographic characteristics encompass age, gender, body mass index, ethnicity, literacy, pre-retirement occupation, marital status, whether the individual resides alone or with others, per capita monthly income with their spouse, religious affiliation, and urban/rural status. Variables related to physical condition and family relationships included the presence or absence of physical impairments, the ability to perform daily activities independently, the number of chronic illnesses, the amount of time spent going to the nearest hospital, the presence of a family history of mental illness, the presence of stressful events in the family in the last year, spousal relationships, children’s relationships, son-in-law’s relationships, neighborhood relationships, the number of days per week spent with relatives and friends, and the amount of time spent per day in contact with relatives and friends. Lifestyle-related variables included smoking, liquor consumption, beer consumption, dairy consumption, soybean consumption, meat consumption, vegetable or fruit consumption, meal habits, daily sleep time, mahjong activity, poker or chess, playing with pets, square dance activity, Tai Chi activity, brisk walking activity, and high-intensity physical activity, household activities, television viewing, and mobile phone use. This study examined 67 variables to identify factors underlying the heterogeneity of mental health among older adults. Supplementary Material 2, Supplemental Digital Content 2 presents the coding process for all study variables.

### 2.4. Statistical analysis

This study analyzed data using 4 software programs: Microsoft Excel 2019, Mplus 8.3, IBM SPSS Statistics 29.0, and RStudio 4.3.1. Statistical significance was defined as *P* < .05 (two-sided tests). In addition, prior to conducting the multivariate logistic regression, all variables that were significant in the univariate analyses were assessed for multicollinearity using the variance inflation factor (VIF). A VIF value of <10 was considered to indicate no significant multicollinearity.

### 2.5. Data preprocessing

Data were organized, screened, and coded using Microsoft Excel 2019. Figures and tables were also generated with Excel.

### 2.6. LCA

An exploratory LCA was conducted using Mplus 8.3. The PHQ-9 and GAD-7 item responses were first dichotomized and then used as categorical indicators to estimate the underlying latent classes. The process involves 3 steps.^[32]^ Initially, the initial model is assumed to be 1 category. Subsequently, the number of categories in the model gradually increases, and the optimal model is selected based on model fitness metrics. Once the optimal number of potential categories had been determined, older adults were assigned to the most likely subgroups based on their highest probability of scoring on each item. Finally, the different categories were named by comparing the observations assigned to the corresponding potential categories.

Metrics used for LCA model fitting include Log-Likelihood (LL), Akaike’s information criterion (AIC),^[33]^ Bayesian information criterion (BIC),^[34]^ sample-corrected BIC (aBIC),^[34]^ entropy,^[35]^ Lo-Mendell–Rubin test (LMR),^[36]^ and bootstrap-based likelihood ratio values (BLRT).^[32]^ The methodology employed to ascertain each indicator is as follows: LL: Larger values represent better performance in model fitting; AIC, BIC, and aBIC: A lower value indicates a superior fit of the model^[37]^; Entropy: A metric of classification accuracy, ranging from 0 to 1; the closer to 1, the higher the classification accuracy of the model. A commonly used threshold for class separation is an entropy value >0.8, suggesting meaningful classification^[19]^; LMR and BLRT: They are indicators of the difference in fit for comparing the models, and if the *P*-value reaches significance, it means that the model with k categories is better than the model with k-1 categories. A *P*-value < .001 was considered statistically significant.^[24]^

### 2.7. Univariate and multivariate analysis

This study conducted the chi-square test and multivariate logistic regression using the SPSS 29.0 software. Chi-square test and Fisher’s exact probability method were used to analyze the factors influencing the differences in mental health levels among older adults. Subsequently, the statistically significant factors identified in the univariate analyses were incorporated into multivariate logistic regression models to ascertain the independent risk factors influencing mental health disparities among older adults. The dependent variable in this study was the potential category subgroup.

### 2.8. Results visualization

Given the considerable number of variables examined in this study, a forest plot of the multivariate logistic regression results was constructed using RStudio version 4.3.1 to facilitate the identification of the independent risk and protective factors influencing the mental health of older adults.

## 3. Results

### 3.1. Demographic characteristics of the elderly aged 65 and above in Guizhou Province

The analysis of 27,880 older adults revealed a predominantly female sample (aged 65–79), of Han ethnicity, with primarily elementary education, monthly incomes below ¥3000, and occupations concentrated in agriculture, forestry, animal husbandry, fishery, and water conservancy. It is noteworthy that the elderly population of the province exhibits a 25.4% prevalence of developing dementia, a 4.4% prevalence of developing depressive symptoms, and a 2.2% prevalence of developing anxiety symptoms. Table 2 shows the demographic characteristics of the sample.

**Table 2:** Demographic and clinical characteristics of the study participants (N = 27,880).

Variable	Categorical variable	Unit (of measure)	Total (N = 27880)	Percentage (%)
Age	65–79	Yr	22,961	82.4
	≥80		4919	17.6
Gender	Male	None	12,599	45.2
	Female		15,281	54.8
BMI	<18.5	Kg/m^2^	1800	6.5
	18.5–25		19,260	69.1
	25–30		5953	21.4
	≥30		867	3.1
Ethnic group	Han Chinese	None	21,962	78.8
	Minority		5918	21.2
Educational level	Below elementary school (illiterate)	None	14,387	51.6
	Elementary education		8975	32.2
	Secondary education (middle school, high school, junior college)		4168	14.9
	Higher education (college or university, university and above)		350	1.3
Occupation before retirement	Unemployed, domestic	None	7019	25.2
	Agricultural, forestry, fishery, water conservancy producers		10,194	36.6
	Business, service industry personnel, clerical staff, related personnel		1014	3.6
	Heads of state organs, party organizations, enterprises, institutions		846	3
	Professionals, technicians		917	3.3
	Operators of production, transportation equipment, related personnel		633	2.3
	Other occupations		7257	26
Marital status	Married	None	21,030	75.4
	Other (divorced, widowed, unmarried)		6850	24.6
Whether living alone	Yes	None	3444	12.4
	Not		24,436	87.6
Whether living with spouse or partner	Yes	None	16,956	60.8
	Not		10,924	39.2
Whether living with grandchildren	Yes	None	2885	10.3
	Not		24,995	89.7
Whether living with other relatives	Yes	None	73	0.3
	Not		27,807	99.7
Whether living with a nursing home partner	Yes	None	26	0.1
	Not		27,854	99.9
Whether living with children	Yes	None	13,730	49.2
	Not		14,150	50.8
Per capita monthly income with spouse	<3000	RMB	26,587	95.4
	3000–6999		1293	4.6
Religious or not	Yes	None	434	1.6
	Not		27,446	98.4
Urban/Rural	Urban	None	16,160	58
	Rural		11,720	42
Depressive symptoms score	0–5	Point	26,657	95.6
	≥5		1223	4.4
Anxiety symptom score	0–5	Point	27,280	97.8
	≥5		600	2.2
Dementia score	0–1	Point	20,808	74.6
	≥2		7072	25.4

### 3.2. Indicators of LCA model fit

**Figure 2.**
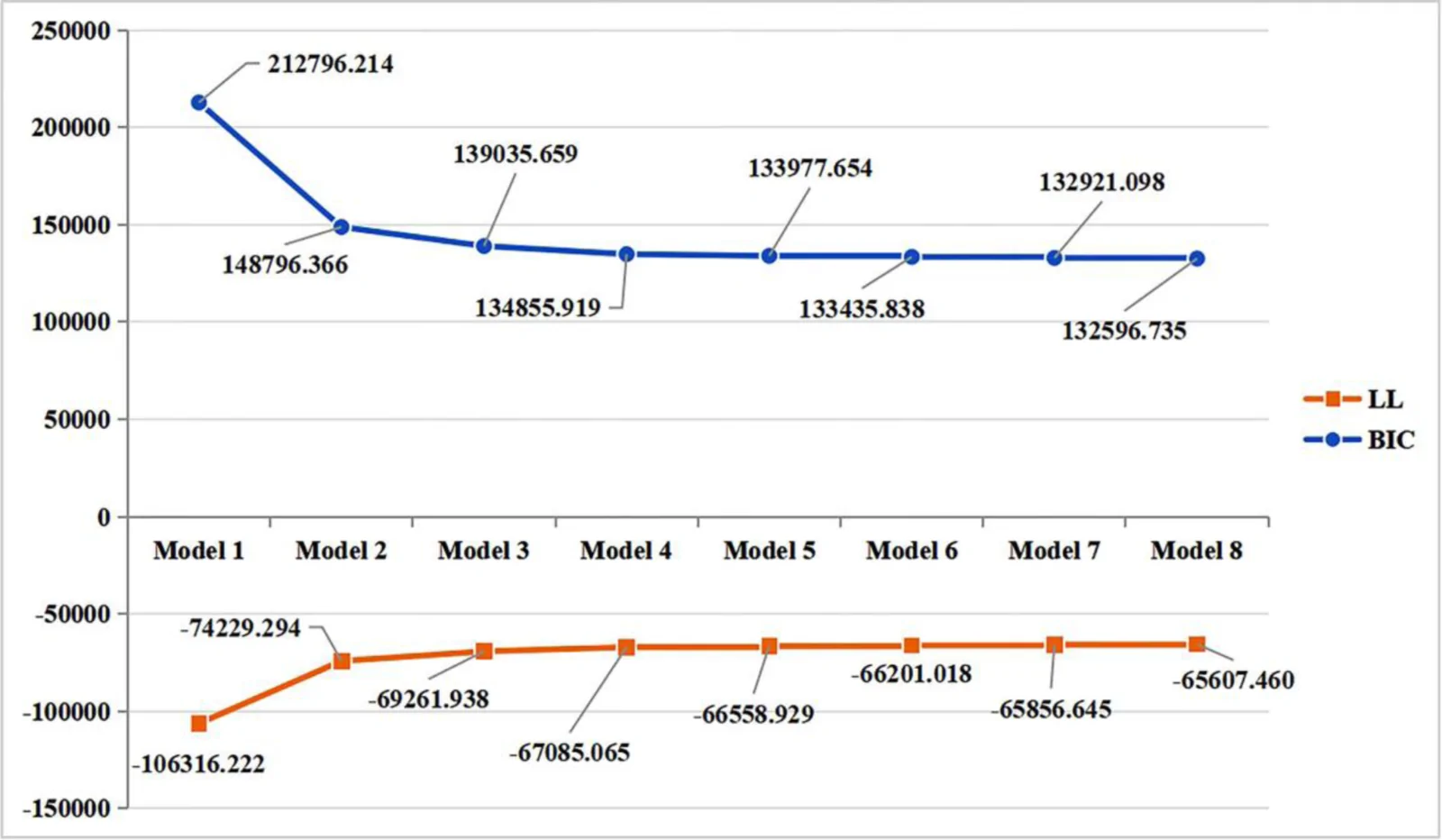
Indicator plots of potential category fit. This figure illustrates the trajectory of key model fit indices across latent class models with one to 8 classes. The X-axis represents the number of latent classes (k) specified in the model. The left Y-axis represents the value of the LL, which increases with model complexity. The right Y-axis represents the value of the BIC, where lower values indicate a better balance between model fit and parsimony. The leveling-off of the LL curve and the clear “elbow” or point of diminishing returns in the descending BIC curve after k = 4 were key visual indicators, in combination with statistical tests (LMR, BLRT) and entropy values (see Table 3), that supported the selection of the 4-class model as the optimal solution for the data. LL = log-likelihood, BIC = Bayesian information criterion, LMR = Lo-Mendell–Rubin test, BLRT = bootstrap-based likelihood ratio.

Table 3 presents the fit indices for the 8 distinct potential categories of models. In order to conduct a comprehensive evaluation of the selected model, we plotted the trajectory of the LL and BIC values, as illustrated in Figure 2. The results demonstrate that the LL values gradually increase with the addition of new categories, whereas the AIC, BIC, and aBIC values demonstrate a declining trend. The AIC, BIC, and aBIC values of the Class 1 model reached their maximum value, indicating that the model exhibited the poorest fit to the data. The entropy value of the Type 2 model is the highest, yet the LMR test result is not statistically significant. As illustrated in Figure 2, following the Type 4 model, the LL and BIC values exhibit slight smoothness. Moreover, higher entropy values indicate more effective category separation.^[35]^ The LMR and BLRT values were statistically significant (*P* < .001) for Class 3 and 4 models. However, the entropy value of the Class 4 model (entropy = 0.904) was slightly higher than that of the Class 3 model (entropy = 0.901), indicating that the Class 4 model exhibited a superior fit to the data compared to the Class 3 model. The Class 5 model demonstrated a lower level of accuracy, with the number of individuals falling into multiple categories representing <5% of the total population. Given the above, we have chosen to use a simple Type 4 model.

**Table 3:** Indicators of fitness for different potential categories of models.

Model	LL	AIC	BIC	ABIC	Entropy	BLRT (*P*-value)	LMR (*P*-value)	Category probability (%)
1	−106316.222	212664.443	212796.214	212745.366				
2	−74229.294	148524.589	148796.366	148691.492	0.958	<.001	.3333	3343 (11.99%)/ 24537 (88.01%)
3	−69261.938	138623.876	139035.659	138876.760	0.901	<.001	<.001	4094 (14.68%)/ 1802 (6.46%)/ 21,984 (78.85%)
4	−67085.065	134304.130	134855.919	134642.994	0.904	<.001	<.001	406 (1.46%)/ 1834 (6.58%)/ 3789 (13.59%) / 21,851 (78.38%)
5	−66558.929	133285.858	133977.654	133710.704	0.910	<.001	<.001	3860 (13.85%)/ 409 (1.47%)/ 475 (1.70%) / 1365 (4.90%)/ 21,771 (78.09%)
6	−66201.018	132604.036	133435.838	133114.862	0.900	<.001	.0054	370 (1.33%)/ 2614 (9.38%)/ 502 (1.80%) / 1190 (4.27%)/ 1150 (4.12%)/ 22054 (79.10%)
7	−65856.645	131949.289	132921.098	132546.096	0.890	<.001	.0012	1021 (3.66%)/ 580 (2.08%)/ 1169 (4.19%) / 372 (1.33%)/ 21,878 (78.47%)/ 505 (1.81%) / 2355 (8.45%)
8	−65607.460	131484.920	132596.735	132167.707	0.894	<.001	.2071	894 (3.21%)/ 532 (1.91%)/ 1024 (3.67%) / 366 (1.31%)/ 21,959 (78.76%)/ 496 (1.78%) / 2257 (8.10%)/ 352 (1.26%)

aBIC = sample-size adjusted BIC, AIC = Akaike information criterion, BIC = Bayesian information criterion, BLRT = bootstrap likelihood ratio test, LL = log-likelihood, LMR = Lo-Mendell-Rubin test.

### 3.3. Definitions of LCA

**Figure 3.**
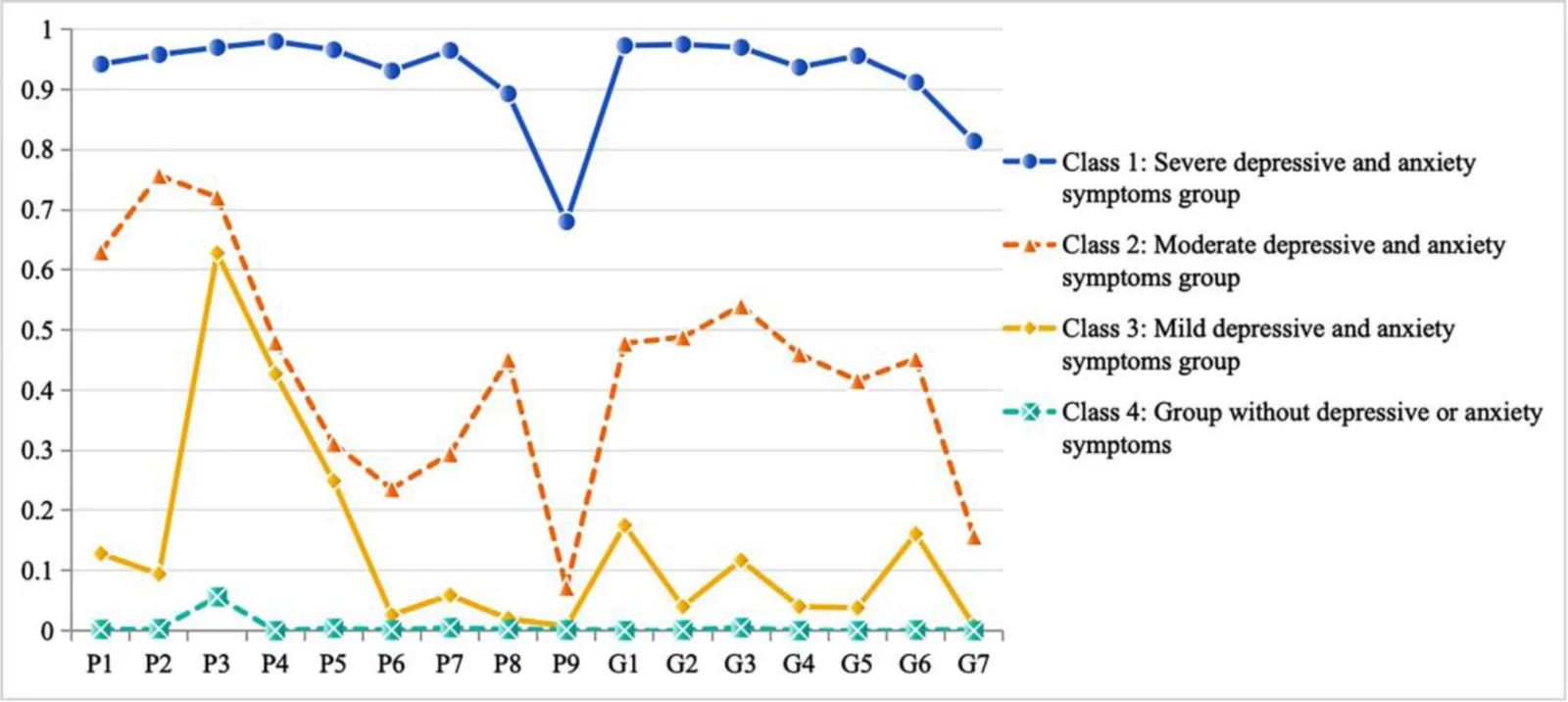
Overview of the potential categories. This figure illustrates the item-response probabilities, which form the basis for interpreting and naming the 4 latent classes. The X-axis lists the 16 indicator variables: the 9 items from the PHQ-9 (P1 to P9) followed by the 7 items from the GAD-7 (G1 to G7). For specific details of the item, please refer to Supplementary Material 1, Supplemental Digital Content 1. The Y-axis represents the probability (ranging from 0 to 1) that an individual within a given latent class would endorse that specific symptom (i.e., score it as present).

Figure 3 illustrates the probability of responding to the entries on the Anxiety Scale and Depression Scale for each category of older adults in the Type 4 model. The horizontal coordinates represent the corresponding entries on the scales, while the vertical coordinates represent the probability of responding. The 4 dotted lines in the figure represent different subgroups of anxiety and depressive symptoms in older adults. Observing that there is no overlap between these 4 lines suggests that the subtypes delineated by the model exhibit significant differences across the symptom spectrum.^[37]^ Notably, of the 4 subtypes, the P4 entry “feeling tired or lacking energy” had the highest probability of response.

Data from Table 3 and Figure 3 indicate that Category 1 (Class 1, N = 406, 1.46%), the smallest group, was more likely to score highly across all assessed domains than other categories. This distinct subgroup of elderly individuals experiences co-occurring anxiety and depressive symptoms, potentially reflecting impaired self-regulation, and is therefore termed the “Severe depressive and anxiety symptoms group.” Category 2 (Class 2, N = 1834, 6.58%) is designated as the “Moderate depressive and anxiety symptoms group” due to its moderately lower scoring on depression and anxiety symptoms in comparison to Class 1. This suggests that this group of older adults possess some capacity for self-regulation. The response rates for anxiety and depression were lower for older adults in Category 3 (Class 3, N = 3789, 13.59%) than for those in the previous 2 categories. This indicated they had superior mental health and could regulate their emotions effectively. Consequently, they were categorized as the “Mild depressive and anxiety symptoms group.” The response rate for depressive and anxiety symptoms among older adults in Category 4 (Class 4, N = 21,851, 78.38%) was almost zero. This category had the largest sample-size. Class 4 was defined as the “Group without depressive or anxiety symptoms.”

### 3.4. Univariate analysis of mental health status in different potential category groups

Chi-square tests assessed associations between the 67 variables and mental health subgroups (Tables 4, 5 and 6). No significant differences emerged for residing with relatives, family history of dementia, or daily beer consumption (*P* > .05). However, significant differences (*P *< .05) were found for variables including age, gender, body mass index, ethnicity, and literacy. Furthermore, statistically significant differences (*P *< .001) in PHQ-9 and GAD-7 scores across subgroups supported the effectiveness of the LCA in differentiating individuals based on mental health status.

**Table 4:** Univariate analysis of variables related to demographic characteristics.

Variable	Unit (of measure)	Categorical variable	Class 1 (N = 406)	Class 2 (N = 1834)	Class 3 (N = 3789)	Class 4 (N = 21,851)	Test statistic	*P*-value
Age	Yr	65–79	312 (76.80%)	1437 (78.40%)	2993 (79.00%)	18,219 (83.40%)	73.93	<.001
		≥80	94 (23.20%)	397 (21.60%)	796 (21.00%)	3632 (16.60%)		
Gender	None	Male	160 (39.40%)	785 (42.80%)	1623 (42.80%)	10,031 (45.90%)	22.713	<.001
		Female	246 (60.60%)	1049 (57.20%)	2166 (57.20%)	11,820 (54.10%)		
BMI	Kg/m^2^	<18.5	32 (7.90%)	172 (9.40%)	283 (7.50%)	1313 (6.00%)	92.073	<.001
		18.5–25	298 (73.40%)	1317 (71.80%)	2549 (67.30%)	15,096 (69.10%)		
		25–30	64 (15.80%)	280 (15.30%)	815 (21.50%)	4794 (21.90%)		
		≥30	12 (3.00%)	65 (3.50%)	142 (3,70%)	648 (3.00%)		
Ethnic group	None	Han Chinese	171 (42.10%)	1250 (62.80%)	2971 (78.40%)	17,570 (80.40%)	485.082	<.001
		Minority	235 (57.90%)	584 (31.80%)	818 (21.60%)	4281 (19.60%)		
Educational level	None	Below elementary school (illiterate)	250 (61.60%)	1187 (64.70%)	1674 (44.20%)	11,276 (51.60%)	311.295	<.001
		Elementary education	128 (31.50%)	489 (26.70%)	1263 (33.30%)	7095 (32.50%)		
		Secondary education (middle school, high school, junior college)	26 (6.40%)	151 (8.20%)	780 (20.60%)	3211 (14.70%)		
		Higher education (college or university, university and above)	2 (0.50%)	7 (0.40%)	72 (1.90%)	269 (1.20%)		
Occupation before retirement	None	Unemployed, domestic	151 (37.20%)	353 (19.20%)	843 (22.20%)	5672 (26.00%)	726.342	<.001
		Agricultural, forestry, fishery, water conservancy producers	153 (37.70%)	1056 (57.60%)	1148 (33.30%)	7837 (35.90%)		
		Business, service industry personnel, clerical staff, related personnel	3 (0.70%)	22 (1.20%)	291 (7.70%)	698 (3.20%)		
		Heads of state organs, party organizations, enterprises, institutions	2 (0.50%)	33 (1.80%)	96 (2.50%)	715 (3.30%)		
		Professionals, technicians	3 (0.70%)	16 (0.90%)	178 (4.70%)	720 (3.30%)		
		Operators of production, transportation equipment, related personnel	4 (1.00%)	34 (1.90%)	108 (2.90%)	487 (2.20%)		
Marital status	None	Other occupations	90 (22.20%)	320 (17.40%)	1125 (29.70%)	5722 (26.20%)	58.676	<.001
		Married	284 (70.00%)	1406 (76.70%)	2684 (70.80%)	16,656 (76.20%)		
		Other (divorced, widowed, unmarried)	122 (30.00%)	428 (23.30%)	1105 (29.20%)	5195 (23.80%)		
Whether living alone	None	Yes	64 (15.80%)	555 (30.30%)	548 (14.50%)	2277 (10.40%)	638.582	<.001
		Not	342 (84.20%)	1279 (69.70%)	3241 (85.50%)	19,574 (89.60%)		
Whether living with spouse or partner	None	Yes	234 (57.60%)	854 (46.60%)	2280 (60.20%)	13,588 (62.20%)	175.865	<.001
		Not	172 (42.20%)	980 (53.40%)	1509 (39.80%)	8263 (37.80%)		
Whether living with grandchildren	None	Yes	31 (7.60%)	104 (5.70%)	211 (5.60%)	2539 (11.60%)	177.844	<.001
		Not	375 (92.40%)	1730 (94.30%)	3578 (94.40%)	19,312 (88.40%)		
Whether living with other relatives	None	Yes	2 (0.50%)	6 (0.30%)	10 (0.30%)	55 (0.30%)	1.970	.525
		Not	404 (99.50%)	1828 (99.70%)	3779 (99.70%)	21,796 (99.70%)		
Whether living with a nursing home partner	None	Yes	2 (0.50%)	7 (0.40%)	9 (0.20%)	8 (0.00%)	39.315	<.001
		Not	404 (99.50%)	1827 (99.60%)	3780 (99.80%)	21,843 (100%)		
Whether living with children	None	Yes	166 (40.90%)	566 (30.90%)	1664 (43.90%)	11,334 (51.90%)	362.584	<.001
		Not	240 (59.10%)	1268 (69.10%)	2125 (56.10%)	10,517 (48.10%)		
Per capita mo income with spouse	RMB	<3000	405 (99.80%)	1793 (97.80%)	3450 (91.10%)	20,939 (95.80%)	211.359	<.001
		3000–6999	1 (0.20%)	41 (2.20%)	339 (8.90%)	912 (4.20%)		
Religious or not	None	Yes	6 (1.50%)	40 (2.20%)	75 (2.00%)	313 (1.40%)	11.301	.010
		Not	400 (98.50%)	1794 (97.80%)	3714 (98.00%)	21,538 (98.60%)		
Urban/Rural	None	Urban	213 (52.50%)	537 (29.30%)	2022 (53.40%)	13,388 (61.30%)	755.199	<.001
		Rural	193 (47.50%)	1297 (70.70%)	1767 (46.60%)	8463 (38.70%)		
Depressive symptoms score	Point	0–5	0 (0.00%)	1177 (64.20%)	3629 (95.80%)	21,851 (100.00%)	14173.447	<.001
		≥5	406 (100.00%)	657 (35.80%)	160 (4.20%)	0 (0.00%)		
Anxiety symptom score	Point	0–5	10 (2.50%)	1632 (89.00%)	3787 (99.90%)	21,851 (100.00%)	18785.731	<.001
		≥5	396 (97.50%)	202 (11.00%)	2 (0.10%)	0 (0.00%)		
Dementia score	Point	0–1	192 (47.30%)	541 (29.50%)	1768 (46.70%)	18,307 (83.80%)	4665.660	<0.001
		≥2	214 (52.70%)	1293 (70.50%)	2021 (53.30%)	3544 (16.20%)		

Data are presented as n (%). P-values were derived from the Chi-square test and Fisher’s exact probability method. Class 1: Severe depressive and anxiety symptoms group; Class 2: Moderate depressive and anxiety symptoms group; Class 3: Mild depressive and anxiety symptoms group; Class 4: Group without depressive or anxiety symptoms.

**Table 5:** Univariate analysis of variables related to physical condition and family relationships.

Variable	Unit (of measure)	Categorical variable	Class 1 (N = 406)	Class 2 (N = 1834)	Class 3 (N = 3789)	Class 4 (N = 21,851)	Test statistic	*P-value*
Physical defects	None	Yes	95 (23.40%)	289 (15.80%)	499 (13.20%)	1139 (5.20%)	687.007	<.001
		Not	311 (76.60%)	1545 (84.20%)	3290 (86.80%)	20,712 (94.80%)		
Ability to perform daily activities independently	None	Yes	400 (98.50%)	1792 (97.70%)	3698 (97.60%)	21,570 (98.70%)	35.217	<.001
		Not	6 (1.50%)	42 (2.30%)	91 (2.40%)	281 (1.30%)		
Number of chronic diseases	Type	No chronic diseases	141 (0.90%)	998 (6.40%)	1836 (11.80%)	12,620 (80.90%)	257.475	<.001
		1 chronic disease	173 (1.80%)	630 (6.60%)	1466 (15.20%)	7348 (76.40%)		
		≥2 chronic diseases	92 (3.40%)	206 (7.70%)	487 (18.30%)	1883 (70.60%)		
Time spent going to nearest hospital	Minute	<30	127 (31.30%)	624 (34.00%)	2268 (59.90%)	12,064 (55.20%)	502.423	<.001
		30–60	264 (65.00%)	1170 (63.80%)	1440 (38.00%)	9002 (41.20%)		
		>60	15 (3.70%)	40 (2.20%)	81 (2.10%)	785 (3.60%)		
Family history of mental illness	None	Yes	17 (4.20%)	40 (2.20%)	60 (1.60%)	289 (1.30%)	30.962	<.001
		Not	389 (95.80%)	1794 (97.80%)	3729 (98.40%)	21,562 (98.70%)		
Family history of Alzheimer’s	None	Yes	5 (1.20%)	17 (0.90%)	33 (0.90%)	162 (0.70%)	2.411	.492
		Not	401 (98.80%)	1817 (99.10%)	3756 (99.10%)	21,689 (99.30%)		
Whether the family had a stressful event in the last yr	None	Yes	13 (3.20%)	21 (1.10%)	67 (1.80%)	109 (0.50%)	107.472	<.001
		Not	393 (96.80%)	1813 (98.90%)	3722 (98.20%)	21,742 (99.50%)		
Spousal relationship	Point	0–3	6 (1.50%)	39 (2.10%)	62 (1.60%)	256 (1.20%)	1783.977	<.001
		4–7	78 (19.20%)	705 (38.40%)	315 (8.30%)	1755 (8.0%)		
		≥8	322 (79.30%)	1090 (59.40%)	3412 (90.10%)	19,840 (90.80%)		
Child relationship	Point	0–3	12 (3.00%)	41 (2.20%)	44 (1.20%)	196 (0.90%)	1765.874	<.001
		4–7	261 (64.30%)	991 (54.00%)	688 (18.20%)	4128 (18.90%)		
		≥8	133 (32.80%)	802 (43.70%)	3057 (80.70%)	17,527 (80.20%)		
Son-in-law or daughter-in-law relationship	Point	0–3	15 (3.70%)	42 (2.30%)	49 (1.30%)	139 (0.60%)	1694.255	<.001
		4–7	128 (31.50%)	854 (46.60%)	559 (14.80%)	2777 (12.70%)		
		≥8	263 (64.80%)	938 (51.10%)	3181 (84.00%)	18,935 (86.70%)		
Neighboring relation	Point	0–3	4 (1.00%)	36 (2.00%)	46 (1.20%)	146 (0.70%)	1215.233	<.001
		4–7	140 (34.50%)	907 (49.50%)	1178 (31.10%)	4061 (18.60%)		
		≥8	262 (64.50%)	891 (48.60%)	2565 (67.70%)	17,644 (80.70%)		
Number of days per week of contact with relatives and friends	d	0	126 (31.0%)	339 (18.50%)	768 (20.30%)	2587 (11.80%)	549.237	<.001
		1–3	154 (37.90%)	1020 (55.60%)	1625 (42.90%)	9443 (43.20%)		
		4–7	126 (31.00%)	475 (25.90%)	1396 (36.80%)	9821 (44.90%)		
Daily contact time with family and friends	Minute	0–30	342 (84.20%)	1028 (56.10%)	3183 (84.00%)	17,500 (80.40%)	663.834	<.001
		≥30	64 (15.80%)	806 (43.90%)	606 (16.00%)	4351 (19.90%)		

Data are presented as n (%). *P*-values were derived from the Chi-square test and Fisher’s exact probability method. Class 1: Severe depressive and anxiety symptoms group; Class 2: Moderate depressive and anxiety symptoms group; Class 3: Mild depressive and anxiety symptoms group; Class 4: Group without depressive or anxiety symptoms.

**Table 6:** Univariate analysis of lifestyle-related variables.

Variable	Unit (of measure)	Categorical variable	Class 1 (N = 406)	Class 2 (N = 1834)	Class 3 (N = 3789)	Class 4 (N = 21,851)	Test statistic	*P*-value
Number of days per wk smoking	d	0	346 (85.20%)	1547 (84.4%)	2930 (77.30%)	17,519 (80.20%)	84.815	<.001
		1–3	18 (4.40%)	17 (0.90%)	51 (1.30%)	408 (1.90%)		
		4–7	42 (10.3%)	270 (14.7%)	808 (21.30%)	3924 (18.00%)		
Number of cigarettes per d	Root	0	344 (84.70%)	1546 (84.3%)	2926 (77.20%)	17,449 (79.90%)	74.270	<.001
		1–10	43 (10.60%)	223 (12.2%)	662 (17.50%)	3418 (15.60%)		
		11–20	17 (4.20%)	63 (3.40%)	166 (4.40%)	913 (4.20%)		
		>20	2 (0.50%)	2 (0.10%)	35 (0.90%)	71 (0.30%)		
Yr of smoking	Yr	0	343 (84.50%)	1540 (84.00%)	2930 (77.30%)	17,436 (79.80%)	149.381	<.001
		1–5	3 (0.70%)	10 (0.50%)	20 (0.50%)	76 (0.30%)		
		6–10	5 (1.20%)	12 (0.70%)	21 (0.60%)	125 (0.60%)		
		11–20	28 (6.90%)	47 (2.60%)	62 (1.60%)	378 (1.70%)		
		≥21	27 (6.70%)	225 (12.30%)	756 (20.00%)	3836 (17.60%)		
Number of d per wk drinking liquor	d	0	366 (90.10%)	1669 (91.00%)	3286 (86.70%)	19,352 (88.60%)	25.146	<.001
		1–3	14 (3.40%)	47 (2.60%)	146 (3.90%)	759 (3.50%)		
		4–7	26 (6.40%)	118 (6.40%)	357 (9.40%)	1740 (8.00%)		
Amount of white spirit consumed per day	Tael	0	368 (90.60%)	1676 (91.40%)	3283 (86.60%)	19,379 (88.70%)	40.892	<.001
		1–2	31 (7.60%)	137 (7.50%)	380 (10.00%)	1957 (9.00%)		
		2–5	6 (1.50%)	19 (1.00%)	106 (2.80%)	423 (1.90%)		
		>5	1 (0.20%)	2 (0.10%)	20 (0.50%)	92 (0.40%)		
History of white wine	Yr	0	366 (90.10%)	1668 (90.90%)	3281 (86.60%)	19,303 (88.30%)	104.966	<.001
		1–5	5 (1.20%)	16 (0.90%)	18 (0.50%)	110 (0.50%)		
		6–10	6 (1.50%)	10 (0.50%)	26 (0.70%)	155 (0.70%)		
		11–20	18 (4.40%)	35 (1.90%)	43 (1.10%)	301 (1.40%)		
		≥21	11 (2.70%)	105 (5.70%)	421 (11.10%)	1982 (9.10%)		
Number of d per wk with beer	d	0	389 (95.80%)	1793 (97.80%)	3705 (97.80%)	21,593 (98.80%)	69.093	<.001
		1–3	13 (3.20%)	29 (1.60%)	66 (1.70%)	160 (0.70%)		
		4–7	4 (1.00%)	12 (0.70%)	18 (0.50%)	98 (0.40%)		
Beer consumption per day	Tael	0–6	406 (100.00%)	1832 (99.90%)	3776 (99.70%)	21,810 (99.80%)	4.224	.209a
		≥6	0 (0.00%)	2 (0.10%)	13 (0.30%)	41 (0.20%)		
Yr of beer	Yr	0	390 (96.10%)	1805 (98.40%)	3725 (98.30%)	21,624 (99.00%)	53.688	<.001a
		1–5	2 (0.50%)	8 (0.40%)	7 (0.20%)	71 (0.30%)		
		6–10	2 (0.50%)	4 (0.20%)	9 (0.20%)	29 (0.10%)		
		11–20	5 (1.20%)	5 (0.30%)	9 (0.20%)	36 (0.20%)		
		≥21	7 (1.70%)	12 (0.70%)	39 (1.00%)	91 (0.40%)		
Number of d per wk with dairy products	d	0	157 (38.70%)	1110 (60.50%)	1633 (43.10%)	9855 (45.10%)	280.902	<.001
		1–3	166 (40.90%)	488 (26.60%)	1014 (26.80%)	6301 (28.80%)		
		4–7	83 (20.40%)	236 (12.90%)	1142 (30.10%)	5695 (26.10%)		
Number of d per wk with soy products	d	0	28 (6.90%)	113 (6.20%)	456 (12.00%)	2294 (10.50%)	118.591	<.001
		1–3	165 (40.60%)	948 (51.70%)	1477 (39.00%)	8893 (40.70%)		
		4–7	213 (52.50%)	773 (42.10%)	1856 (49.00%)	10,664 (48.80%)		
Number of d per wk of meat consumption	d	0	18 (4.40%)	50 (2.70%)	141 (3.70%)	794 (3.60%)	87.346	<.001
		1–3	153 (37.70%)	344 (18.80%)	885 (23.40%)	5489 (25.10%)		
		4–7	235 (57.90%)	1440 (78.50%)	2763 (72.90%)	15,568 (71.20%)		
Number of d of vegetable or fruit consumption per wk	d	0	15 (3.70%)	45 (2.50%)	97 (2.60%)	734 (3.40%)	184.889	<.001
		1–3	41 (10.10%)	88 (4.80%)	152 (4.00%)	2117 (9.70%)		
		4–7	350 (86.20%)	1701 (92.70%)	3540 (93.40%)	19,000 (87.00%)		
Dining pattern	None	Breakfast, lunch, dinner	271 (66.70%)	751 (40.90%)	2583 (68.20%)	15,680 (71.80%)	857.138	<.001
		Breakfast, lunch, dinner, evening meal	2 (0.50%)	17 (0.90%)	55 (1.50%)	277 (1.30%)		
		Breakfast, lunch	3 (0.70%)	11 (0.60%)	45 (1.20%)	157 (0.70%)		
		Breakfast, dinner	7 (1.70%)	60 (3.30%)	94 (2.50%)	554 (2.50%)		
		Other	123 (30.30%)	995 (54.30%)	1012 (26.70%)	5183 (23.70%)		
Daily sleeping hours	h	<7	4 (1.00%)	33 (1.80%)	90 (2.40%)	383 (1.80%)	338.287	<.001
		7–9	205 (50.50%)	725 (39.50%)	2366 (62.40%)	13,002 (59.50%)		
		>9	197 (48.50%)	1076 (58.70%)	1333 (35.20%)	8466 (38.70%)		
Number of d per wk for mahjong	d	0	389 (95.8%)	1774 (96.70%)	3504 (92.50%)	20,718 (94.80%)	63.674	<.001
		1–3	12 (3.00%)	30 (1.60%)	180 (4.80%)	619 (2.80%)		
		4–7	5 (1.20%)	30 (1.60%)	105 (2.80%)	514 (2.40%)		
Mahjong time per d	h	0	387 (95.30%)	1781 (97.10%)	3512 (92.70%)	20,727 (94.90%)	90.607	<.001
		0–2	13 (3.20%)	26 (1.40%)	70 (1.80%)	454 (2.10%)		
		>2	6 (1.5%)	27 (1.50%)	207 (5.50%)	670 (3.10%)		
Number of poker or chess d a wk	d	0	391 (96.30%)	1767 (96.30%)	3612 (95.30%)	21,078 (96.50%)	22.540	<.001
		1–3	10 (2.50%)	39 (2.10%)	82 (2.20%)	450 (2.10%)		
		4–7	5 (1.20%)	28 (1.50%)	95 (2.50%)	323 (1.50%)		
Daily poker or chess time	h	0	388 (95.60%)	1772 (96.60%)	3619 (95.50%)	21,111 (96.60%)	15.370	.018
		0–2	14 (3.40%)	48 (2.60%)	121 (3.20%)	567 (2.60%)		
		>2	4 (1.00%)	14 (0.80%)	49 (1.30%)	173 (0.80%)		
Number of d per wk playing with pets	d	0	397 (97.80%)	1799 (98.10%)	3709 (97.90%)	21,651 (99.10%)	77.849	<.001
		1–3	7 (1.70%)	15 (0.80%)	19 (0.50%)	66 (0.30%)		
		4–7	2 (0.50%)	20 (1.10%)	61 (1.60%)	134 (0.60%)		
Daily time with pets	Min	0	395 (97.30%)	1801 (98.20%)	3715 (98.00%)	21,651 (99.10%)	54.325	<.001a
		0–15	3 (0.70%)	12 (0.70%)	36 (1.00%)	57 (0.30%)		
		>15	8 (2.00%)	21 (1.10%)	38 (1.00%)	143 (0.70%)		
Number of square dance d per wk	d	0	383 (94.30%)	1762 (96.10%)	3629 (95.80%)	20,965 (95.90%)	76.043	<.001
		1–3	13 (3.20%)	25 (1.40%)	28 (0.70%)	464 (2.10%)		
		4–7	10 (2.50%)	47 (2.60%)	132 (3.50%)	422 (1.90%)		
Daily square dance time	Min	0	384 (94.60%)	1766 (96.30%)	3634 (95.90%)	20,959 (95.90%)	14.487	.025
		0–30	18 (4.40%)	36 (2.00%)	84 (2.20%)	567 (2.60%)		
		>30	4 (1.00%)	32 (1.70%)	71 (1.90%)	325 (1.50%)		
Number of Tai Chi d per wk	d	0	398 (98.00%)	1807 (98.50%)	3742 (98.80%)	21,519 (98.50%)	21.863	.001
		1–3	4 (1.0%)	12 (0.70%)	9 (0.20%)	185 (0.80%)		
		4–7	4 (1.00%)	15 (0.80%)	38 (1.00%)	147 (0.70%)		
Daily Tai Chi Time	Min	0	398 (98.0%)	1818 (99.10%)	3749 (98.90%)	21,538 (98.60%)	25.447	<.001
		0–30	7 (1.70%)	11 (0.60%)	17 (0.40%)	238 (1.10%)		
		>30	1 (0.20%)	5 (0.30%)	23 (0.60%)	75 (0.30%)		
Number of brisk walking d per wk	d	0	372 (91.60%)	1710 (93.20%)	3503 (92.50%)	20,120 (92.10%)	86.682	<.001
		1–3	23 (5.70%)	48 (2.60%)	81 (2.10%)	317 (1.50%)		
		4–7	11 (2.70%)	76 (4.10%)	205 (5.40%)	1414 (6.50%)		
Daily brisk walking time	Min	0	373 (91.90%)	1713 (93.40%)	3504 (92.50%)	20,118 (92.10%)	16.692	.01
		0–30	28 (6.90%)	82 (4.50%)	215 (5.70%)	1163 (5.30%)		
		>30	5 (1.20%)	39 (2.10%)	70 (1.80%)	570 (2.60%)		
Number of d of high-intensity physical activity per wk	d	0	396 (97.50%)	1781 (97.10%)	3685 (97.30%)	21,393 (97.90%)	19.323	.004
		1–3	5 (1.20%)	27 (1.50%)	31 (0.80%)	181 (0.80%)		
		4–7	5 (1.20%)	26 (1.40%)	73 (1.90%)	277 (1.30%)		
Daily high-intensity physical activity time	Min	0	396 (97.50%)	1794 (97.80%)	3702 (97.70%)	21,443 (98.10%)	16.190	.013
		0–15	3 (0.70%)	6 (0.30%)	11 (0.30%)	112 (0.50%)		
		>15	7 (1.70%)	34 (1.90%)	76 (2.00%)	296 (1.40%)		
Number of d of housework per wk	d	0	153 (37.70%)	354 (19.30%)	627 (16.50%)	4937 (22.60%)	176.469	<.001
		1–3	76 (18.70%)	324 (17.70%)	565 (14.90%)	3760 (17.20%)		
		4–7	177 (43.60%)	1156 (63.00%)	2597 (68.50%)	13,154 (60.20%)		
Daily time for household activities	Min	0	153 (37.70%)	378 (20.60%)	677 (17.90%)	5128 (23.50%)	351.106	<.001
		0–30	183 (45.10%)	1121 (61.10%)	1957 (51.60%)	9871 (45.20%)		
		30–60	46 (11.30%)	244 (13.30%)	764 (20.20%)	5077 (23.20%)		
		>60	24 (5.90%)	91 (5.00%)	391 (10.30%)	1775 (8.10%)		
Number of television d per wk	d	0	201 (49.50%)	399 (21.80%)	750 (19.80%)	4764 (21.80%)	1450.185	<.001
		1–3	47 (11.60%)	714 (38.90%)	368 (9.70%)	2457 (11.20%)		
		4–7	158 (38.90%)	721 (39.30%)	2671 (70.50%)	14,630 (67.00%)		
Daily television time	Min	0	202 (49.80%)	399 (21.80%)	759 (20.00%)	4776 (21.90%)	890.699	<.001
		0–30	123 (30.30%)	821 (44.80%)	715 (18.90%)	5514 (25.20%)		
		30–60	60 (14.80%)	331 (18.00%)	966 (25.50%)	6271 (28.70%)		
		>60	21 (5.20%)	283 (15.40%)	1349 (35.60%)	5290 (24.20%)		
Number of d per wk using cell phones	D	0	274 (67.50%)	657 (35.80%)	1476 (39.00%)	8624 (39.50%)	835.303	<.001
		1–3	45 (11.10%)	760 (41.40%)	615 (16.20%)	3872 (17.70%)		
		4–7	87 (21.40%)	417 (22.70%)	1698 (44.80%)	9355 (42.80%)		
Time spent using cell phone per d	Min	0	291 (71.70%)	709 (38.70%)	1640 (43.30%)	9705 (44.40%)	164.186	<.001
		0–30	108 (26.60%)	1111 (60.60%)	2094 (55.30%)	11,937 (54.60%)		
		>30	7 (1.70%)	14 (0.80%)	55 (1.50%)	209 (1.00%)		

Data are presented as n (%). *P*-values were derived from the Chi-square test and Fisher’s exact probability method. Class 1: Severe depressive and anxiety symptoms group; Class 2: Moderate depressive and anxiety symptoms group; Class 3: Mild depressive and anxiety symptoms group; Class 4: Group without depressive or anxiety symptoms. “a” represents Fisher’s exact probability.

### 3.5. Multivariate logistic regression of mental health status of different potential category groups

Table 7 illustrates the information fitting the multiple logistic regression model. The likelihood ratio test for the model was statistically significant (*P* < .001), indicating a significant overall fit. To address potential multicollinearity among the independent variables, we calculated VIFs for all covariates included in the multivariable logistic regression models. Two variables, weekly smoking days (VIF = 12.22) and years of smoking (VIF = 14.376), exhibited VIFs above 10, suggesting correlation between these factors. Given their theoretical importance in mental health research, we retained these variables in the final model. The overall model performance and interpretation were not substantially affected, as evidenced by the consistent results across other covariates.

**Table 7:** Model fit information.

Likelihood ratio test		Chi-square	*P*-value
Intercept only	39116.112		
Eventual	29053.777	10062.335	<.001

**Figure 4.**
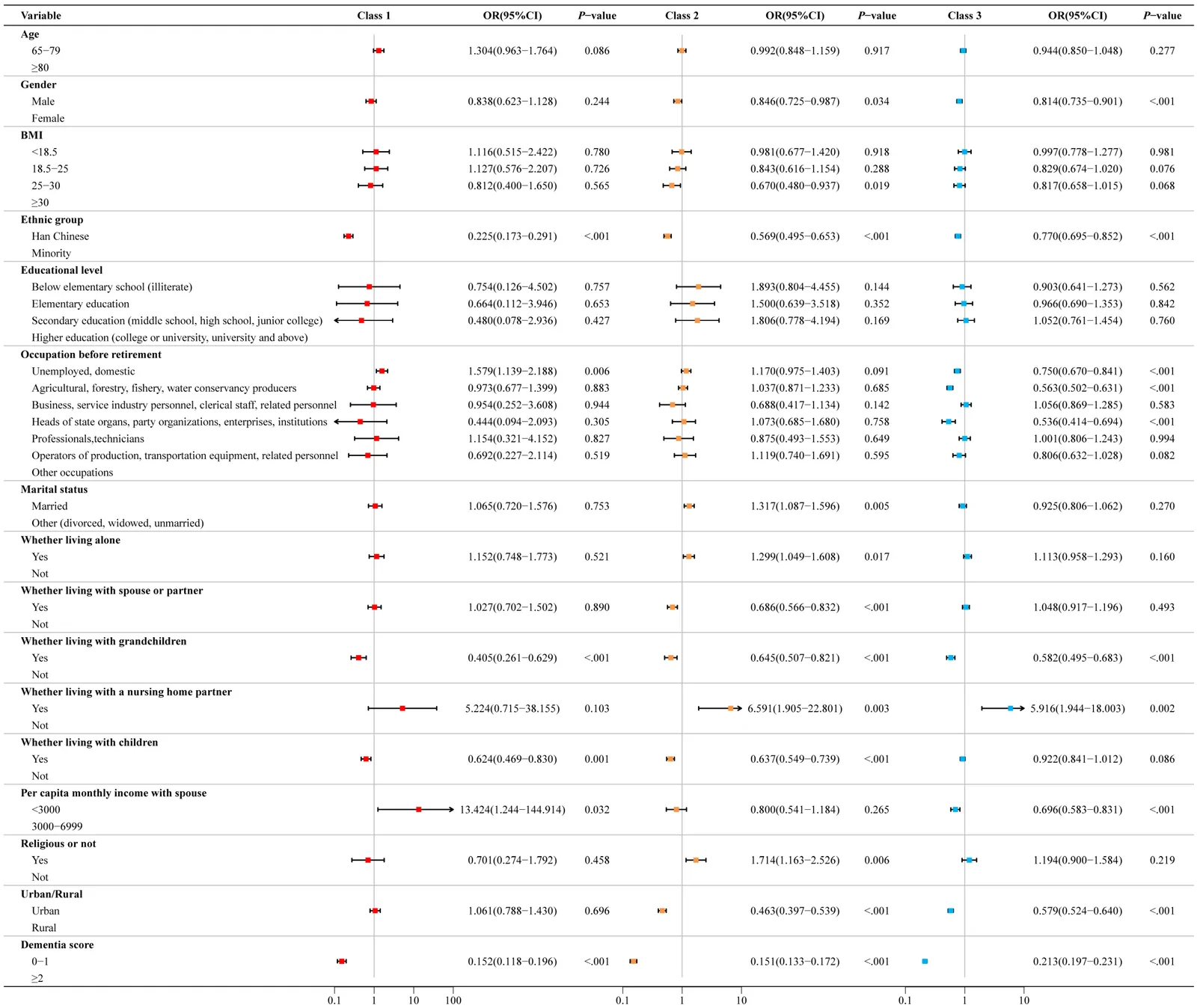
Multiple logistic regression analysis of variables related to demographic characteristics. This forest plot displays the odds ratios (ORs) and their 95% confidence intervals (95% CIs) from the multivariate logistic regression analysis, with the asymptomatic group (Class 4) as the reference. The X-axis represents the odds ratio on a logarithmic scale. An OR > 1 indicates a higher likelihood of belonging to the specified symptomatic class (Class 1, 2, or 3), while an OR < 1 indicates a protective factor for membership in the asymptomatic class. The Y-axis lists the independent variables included in the model. Each point represents the point estimate of the OR, and the horizontal lines represent the 95% CI. A CI that does not cross the vertical dashed line at OR = 1 indicates a statistically significant association (*P* < .05). CI = confidence intervals, OR = odds ratios.

**Figure 5.**
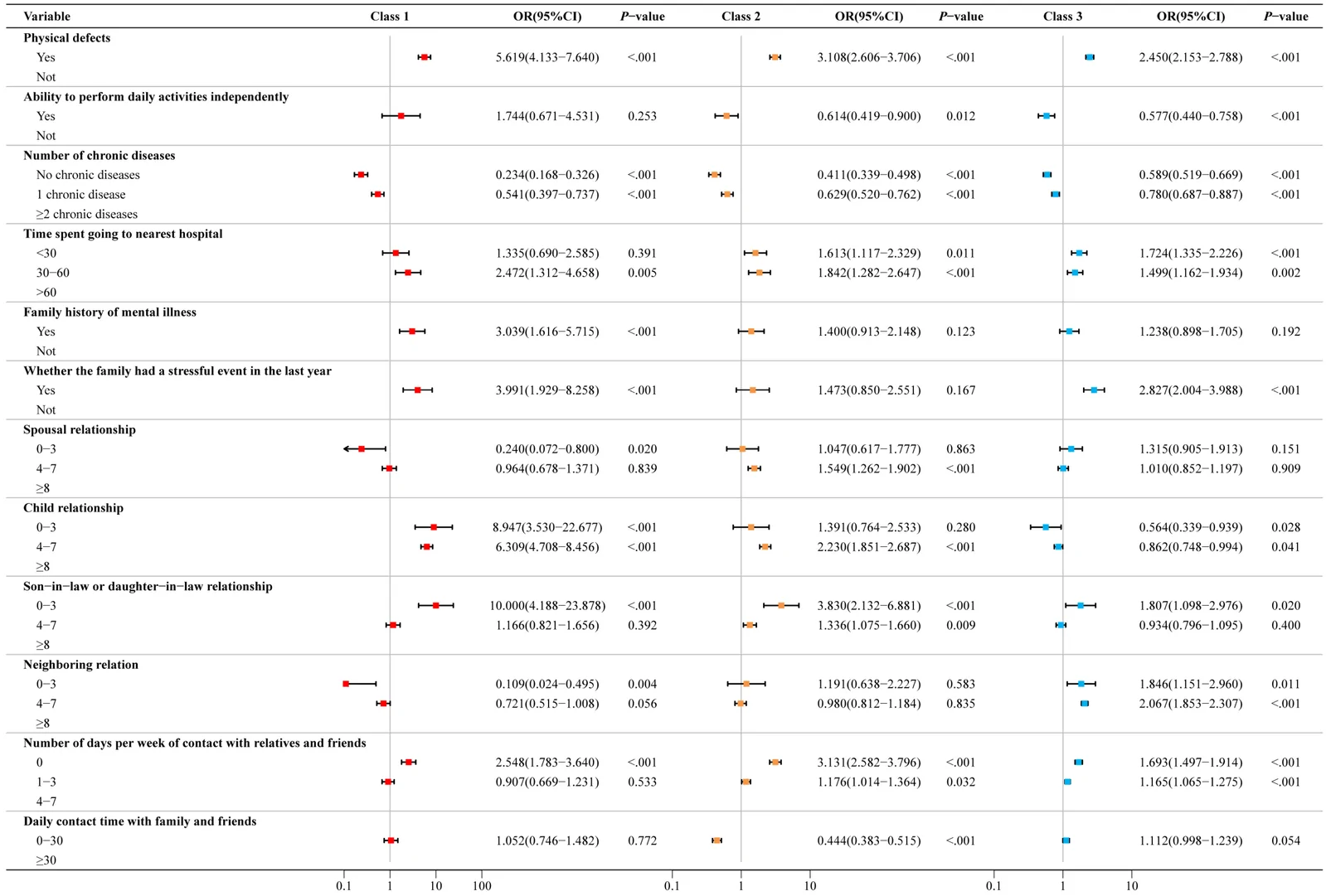
Multiple logistic regression analyses of variables related to physical condition and family relationships. This forest plot displays the odds ratios (ORs) and their 95% confidence intervals (95% CIs) from the multivariate logistic regression analysis, with the asymptomatic group (Class 4) as the reference. The X-axis represents the odds ratio on a logarithmic scale. An OR > 1 indicates a higher likelihood of belonging to the specified symptomatic class (Class 1, 2, or 3), while an OR < 1 indicates a protective factor for membership in the asymptomatic class. The Y-axis lists the independent variables included in the model. Each point represents the point estimate of the OR, and the horizontal lines represent the 95% CI. A CI that does not cross the vertical dashed line at OR = 1 indicates a statistically significant association (*P* < .05). CI = confidence intervals, OR = odds ratios.

**Figure 6.**
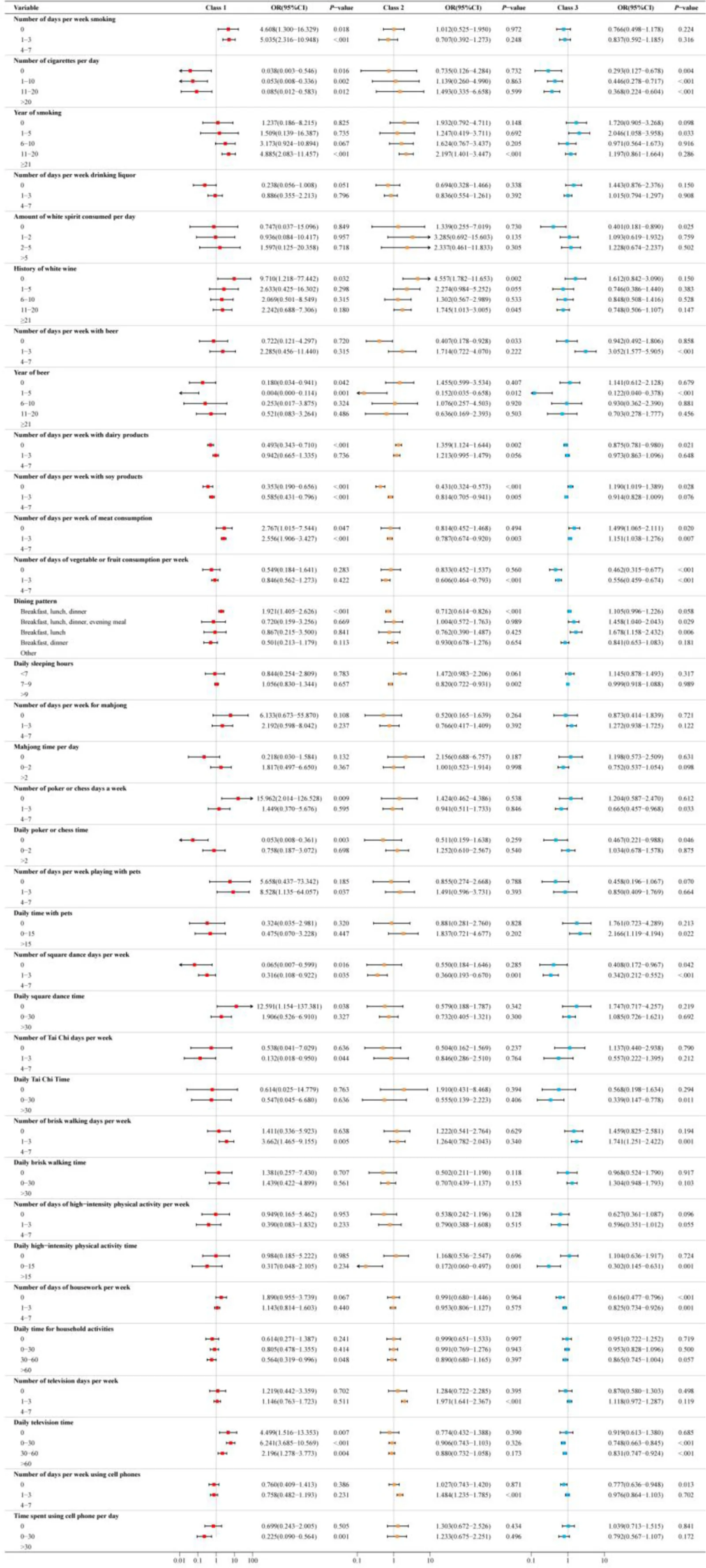
Multiple logistic regression analysis of lifestyle-related variables. This forest plot displays the odds ratios (ORs) and their 95% confidence intervals (95% CIs) from the multivariate logistic regression analysis, with the asymptomatic group (Class 4) as the reference. The X-axis represents the odds ratio on a logarithmic scale. An OR > 1 indicates a higher likelihood of belonging to the specified symptomatic class (Class 1, 2, or 3), while an OR < 1 indicates a protective factor for membership in the asymptomatic class. The Y-axis lists the independent variables included in the model. Each point represents the point estimate of the OR, and the horizontal lines represent the 95% CI. A CI that does not cross the vertical dashed line at OR = 1 indicates a statistically significant association (*P* < .05). CI = confidence intervals, OR = odds ratios.

Four subgroups served as dependent variables, with the fourth as the reference. All variables significant in the univariate analysis were entered into a multivariate logistic regression model to identify independent factors associated with latent class membership. The complete and detailed outputs of this regression analysis, including odds ratios (ORs), confidence intervals, and *P*-values for every variable across all class comparisons, are available in Supplementary Materials 3, 4, and 5. To facilitate the interpretation of these extensive results, we distilled the key findings into forest plots (Figures 4, 5, and 6). For instance, Supplementary Material 3 (Supplemental Digital Content 3) and Figure 4 focus on demographic factors, revealing that urban residence and co-residing with family were strong protective factors, while pre-retirement unemployment was a significant risk factor. Similarly, the patterns for physical health and family relationships are detailed in Supplementary Material 4 (Supplemental Digital Content 4) and visualized in Figure 5, and the extensive analysis of lifestyle behaviors is available in Supplementary Material 5 (Supplemental Digital Content 5) and Figure 6. To summarize the most salient findings, the factors exhibiting the strongest and most consistent associations across subgroups are highlighted here. Key independent risk factors for belonging to the more severe symptom classes (Class 1 or 2) included: the presence of physical disability, a history of family mental illness, poor relationships with children or in-laws, and the absence of weekly social contact. Conversely, major protective factors associated with the asymptomatic group (Class 4) included: independent activities of daily living, the absence of chronic diseases, co-residence with family (spouse or children), and regular consumption of vegetables and fruits. A synthesis of these pivotal findings and their public health implications is presented in Table 8.

**Table 8:** Core factors influencing anxiety and depression in older adults.

Domain	Key factor	Association	Proposed public health intervention strategy
Demographic & social	Living alone	Risk	Develop community-based “buddy systems” and promote participation in senior activity centers to reduce social isolation.
	Urban residence	Protective	Explore strategies to extend urban-based mental health resources and social support models to rural areas.
	Low household income	Risk	Advocate for the strengthening of social pensions and targeted subsidy programs for impoverished elderly.
Physical Health	Physical disability	Risk	Integrate physical rehabilitation services with psychological screening and support in primary care settings.
	Multiple chronic diseases	Risk	Implement collaborative care models that manage chronic conditions while concurrently addressing mental health.
	Independent ADL	Protective	Support programs that help maintain functional independence through home safety modifications and assistive devices.
Family & social	Poor family relationships	Risk	Offer family mediation services and psychoeducation for relatives on supporting elderly mental health.
	No wk social contact	Risk	Establish community group activities (e.g., tea gatherings, hobby clubs) facilitated by local volunteers.
Lifestyle	Irregular meal patterns	Risk	Promote community “Elderly Canteens” and nutritional education programs to ensure regular, balanced diets.
	Inadequate vegetable/fruit intake	Risk	
	Moderate physical activity (e.g., Tai Chi)	Protective	Subsidize and widely offer group-based exercise programs like Tai Chi or brisk walking in communities.
	Moderate sleep (7–9 hours)	Protective	Launch public health campaigns on sleep hygiene specifically tailored for older adults.

Demographic analysis revealed significant factors associated with subgroups of depressive and anxiety symptoms (Supplementary Material 3, Supplemental Digital Content 3, Figure 4). Older adults were significantly more likely to belong to the group without depressive or anxiety symptoms (Class 4) if they were male (OR = 0.846, 95%CI [0.725–0.987], *P* = .034), overweight (OR = 0.670, 95%CI [0.480–0.937], *P* = .019), of Han Chinese ethnicity (OR = 0.770, 95%CI [0.695–0.852], *P* < .001), living with a spouse (OR = 0.686, 95%CI [0.566–0.832], *P* < .001), grandchildren (OR = 0.645, 95%CI [0.570–0.821], *P* < .001), or children (OR = 0.637, 95%CI [0.549–0.739], *P* < .001), or residing in urban areas (OR = 0.579, 95%CI [0.524–0.640], *P* < .001). Conversely, those unemployed or engaged only in housework before retirement (OR = 1.579, 95%CI [1.139–2.188], *P* = .006) or with a per capita monthly household income less than ¥3000 (OR = 13.424, 95%CI [1.244–144.914], *P* = .032) were more likely to be in the severe depressive and anxiety symptoms group (Class 1). Being married (OR = 1.137, 95%CI [1.087–1.596], *P* < .001), living alone (OR = 1.299, 95%CI [1.049–1.608], *P* = .017), having a religious affiliation (OR = 1.714, 95%CI [1.163–2.526], *P* = .006), or co-residing with a nursing home partner (OR = 6.591, 95%CI [1.905–22.801], *P* = .003) increased the likelihood of classification in the moderate depressive and anxiety symptoms group (Class 2). In addition, the absence of cognitive impairment (Ascertain Dementia 8 score 0–1) was a strong predictor of the absence of depressive or anxiety symptoms in older adults (OR = 0.213, 95%CI [0.197–0.231], *P* = .003).

Analysis of physical condition and family relationships revealed key protective and risk factors (Supplementary Material 4, Supplemental Digital Content 4, Figure 5). Older adults were significantly more likely to belong to the Class 4 if they had independent daily living ability (OR = 0.614, 95%CI [0.419–0.900], *P* = .012), no chronic diseases (OR = 0.589, 95%CI [0.519–0.669], *P* < .001), or only 1 chronic disease (OR = 0.780, 95%CI [0.687–0.887], *P* < .001). Conversely, physical impairment (OR = 5.619, 95%CI [4.133–7.640], *P* < .001), travel time to hospital of 30 to 60 minutes (OR = 2.472, 95%CI [1.312–4.658], *P* = .005), family history of mental illness (OR = 3.039, 95%CI [1.616–5.715], *P* < .001), stressful family events in the past year (OR = 3.991, 95%CI [1.929–8.258], *P* < .001), poor relationships with children (OR = 8.947, 95%CI [3.530–22.677], *P* < .001) or in-laws (OR = 10.000, 95%CI [4.188–23.878], *P* < .001) significantly increased the likelihood of classification in the Class 1. Worse spousal relationships (scores 4–7; OR = 1.549, 95%CI [1.262–1.902], *P* < .001), poorer in-law relationships (scores 0–3; OR = 3.830, 95%CI [2.132–6.881], *P* < .001), and no weekly contact with friends/relatives (OR = 3.131, 95%CI [2.582–3.796], *P* < .001) predicted classification in the Class 2. Shorter hospital travel time (<30 minutes; OR = 1.724, 95%CI [1.335–2.226], *P* < .001) and moderate neighborhood relationship scores (4–7; OR = 2.067, 95%CI [1.853–2.307], *P* < .001) were associated with Class 3.

In the multivariate analysis of lifestyle factors (Supplementary Material 5, Supplemental Digital Content 5, Figure 6), several behaviors were significantly associated with classification into the Class 4. Protective factors included: smoking fewer than 20 cigarettes/day (OR = 0.446, 95%CI [0.278–0.717], *P* < .001), abstaining from liquor (OR = 0.401, 95%CI [0.181–0.890], *P* = .025) or beer (OR = 0.407, 95%CI [0.178–0.928], *P* = .003), consuming soy products (OR = 0.814, 95%CI [0.705–0.941], *P* = .005) or vegetables/fruits (OR = 0.606, 95%CI [0.464–0.793], *P* < .001) ≤ 3 days/week, sleeping 7 to 9 hours/day (OR = 0.820, 95%CI [0.722–0.931], *P* = .002), playing poker/chess ≤3 days/week (OR = 0.665, 95%CI [0.457–0.968], *P* = .033), engaging in square dancing ≤3 days/week (OR = 0.360, 95%CI [0.193–0.670], *P* = .001) or not at all (OR = 0.065, 95% CI [0.007–0.599], *P* = .016), practicing tai chi ≤3 days/week (OR = 0.132, 95%CI [0.018–0.950], *P* = .044) or ≤30 minutes/day (OR = 0.339, 95%CI [0.147–0.778], *P* = .001), performing vigorous activity ≤15 minutes/day (OR = 0.302, 95% CI [0.145–0.631], *P* < .001), doing housework ≤3 days/week (OR = 0.825, 95%CI [0.734–0.926], *P* = .001) or ≤ 1 hour/day (OR = 0.564, 95% CI [0.319–0.996], *P* = .048), and using a cellphone ≤ 30 minutes/day (OR = 0.225, 95%CI [0.090–0.564], *P* = .001) or not at all (OR = 0.777, 95%CI [0.636–0.948], *P* = .013). Compared with Class 4, older adults were significantly more likely to be classified into Class 1 if they were: nonsmokers (OR = 4.608, 95%CI [1.300–16.329], *P* = .018) or smoked 1 to 3 days/week (OR = 5.035, 95% CI [2.316–10.948], *P* < .001); had a smoking history of 11 to 20 years (OR4.885, 95% CI [2.083–11.457], *P* < .001); had consumed alcohol for <1 year (OR = 9.710, 95%CI [1.218–77.442], *P* = .032); consumed meat 0 days/week (OR = 2.767, 95%CI [1.015–7.544], *P* = .047) or 1 to 3 days/week (OR = 2.556, 95%CI [1.906–3.427], *P* = .047); maintained daily breakfast, lunch, and dinner habits (OR = 1.921, 95%CI [1.405–2.626], *P* < .001); played poker/chess < 1 day/week (OR = 15.962, 95%CI [2.014–126.528], *P* = .009); played with pets ≤3 days/week (OR = 8.528, 95%CI [1.135–64.057], *P* = .037); engaged in square dancing ≤30 minutes/day (OR = 12.591, 95%CI [1.154–137.381], *P* = .038); engaged in brisk walking ≤3 days/week (OR = 3.662, 95%CI [1.465–9.155], *P* = .005); or watched television <1 hour/day (OR = 2.196, 95% CI [1.278–3.773], *P* = .004). Compared with Class 4, older adults were more likely to be classified into Class 2 if they had: 11 to 20 years of liquor consumption (OR = 1.745, 95%CI [1.013–3.005], *P* = .045), no weekly dairy intake (OR = 1.359, 95%CI [1.124–1.644], *P* = .002), watched television 1 to 3 days/week (OR = 1.971, 95%CI [1.641–2.367], *P* < .001), or used cell phones 1 to 3 days/week (OR = 1.484, 95% CI [1.235–1.785], *P* < .001). Similarly, compared with Class 4, individuals were more likely to be classified into Class 3 if they had: 1 to 5 years of smoking history (OR = 2.046, 95%CI [1.058–3.958], *P* = .033), consumed beer ≤3 days/week (OR = .052, 95%CI [1.577–5.905], *P* < .001), consumed no soy products weekly (OR = 1.190, 95%CI [1.019–1.389], *P* = .028), maintained specific meal patterns (breakfast + lunch: OR = 1.678, 95%CI [1.158–2.432], *P* = .006; breakfast + lunch + dinner snacks: OR = 1.458, 95%CI [1.040–2.043], *P* = .029), or played with pets <15 minutes/day (OR = 2.166, 95% CI [1.119–4.194], *P* = .022).

## 4. Discussion

### 4.1. Group homogeneity of depressive and anxiety symptoms in people aged 65 and over

This study is the first to use LCA to classify and analyze psychological research data on older adults collected by the Guizhou Provincial Center for Disease Control and Prevention. The observed prevalence of depressive and anxiety symptoms in Guizhou Province was higher than that reported by Zhenfan He et al in a Guangdong-based study of adults aged 60 and above (2.19% for depressive symptoms and 1.39% for anxiety symptoms).^[38]^ Additionally, the prevalence rate of cognitive impairment was also higher than the national average (6%).^[39]^ This discrepancy may be attributed to the relative economic backwardness, inadequate social support, and prevalent medical conditions in Guizhou Province. Guangdong Province has a more comprehensive mental health service system, effectively enhancing the mental health of the elderly. Economically disadvantaged regions must prioritize mental health education, establish urban-rural service networks, and strengthen professional capacity to address aging challenges.

The latent class model yielded a 4-class model, establishing distinct symptom profiles of depression and anxiety in adults aged 65 and older. The “severe depressive and anxiety symptoms group,” though the smallest, presents the most severe conditions and requires focused attention. Key characteristics include pre-retirement unemployment, low-income, weak social support, and unhealthy lifestyles. Healthcare workers must implement tailored interventions addressing these unique needs to reduce symptoms and improve cognitive and overall health. The “moderate depressive and anxiety symptoms group” shows a trend towards higher symptom severity. Although current economic status, social support, and physical fitness provide some stability, these individuals have limited coping capacity and may develop pessimism, increasing their risk for depression and anxiety. Healthcare professionals should closely monitor symptoms and implement timely interventions to prevent deterioration. The “mild depressive and anxiety symptoms group” exhibited good overall mental health with only mild symptoms. This is likely due to good physical functioning, strong social support, frequent interactions, higher education, and better cognition. Notably, most older adults with depressive and anxiety symptoms in Guizhou fall into this group, reflecting a generally low-symptom state. Implementing early, individualized interventions for this group could significantly improve mental health, reduce suicide risk, and lessen societal burden. As many as 78.3% of elderly patients were classified as “group without depressive or anxiety symptoms,” indicating that this group of elderly patients experienced minimal psychological stress. This may be related to the low detection rate of depressive and anxiety symptoms among the elderly in this survey.

### 4.2. The impact of demographic characteristics on depressive and anxiety symptoms in older adults

Identified protective factors for mental health in older adults included overweight, Han ethnicity, certain pre-retirement occupations (e.g., technician, farmer), co-residence with family members (such as a spouse or grandchildren), urban residence, and absence of cognitive impairment. In community-dwelling older adults, maintaining a body weight within or above the ideal range may be associated with better mental health outcomes.^[40]^ Ethnic minority older adults have been shown to face a heightened risk of depression,^[41]^ while robust social support networks, including spouses, children, grandchildren, and other relatives or friends, play a significant role in mitigating depression and anxiety, consistent with existing literature.^[42]^ Retirement has been linked to worsening depressive symptoms, particularly among men from lower occupational backgrounds.^[43]^ Older adults in urban settings generally exhibit lower rates of depressive and anxiety symptoms than their rural counterparts,^[44]^ likely due to the multiple challenges prevalent in rural areas, such as lower educational attainment, poorer health status, loneliness, weaker social support structures, limited healthcare access, fewer recreational options, and economic precarity: all of which may diminish coping capacity and increase vulnerability to depression and anxiety. Furthermore, evidence supports a consistent association between cognitive impairment and symptoms of anxiety and depression.^[45,46]^ As demonstrated by Lei Jin et al cognitive impairment increases the risk of depressive symptoms by 1.206-fold.^[46]^ Impaired cognitive function adversely affects memory, comprehension, and activities of daily living, often leading to disorientation, social isolation, and communication difficulties, which may in turn intensify feelings of loneliness and further elevate the risk of anxiety and depression.

Independent risk factors for elderly psychological problems included: married status, living alone, nursing home residence, spouse per capita income <¥3000/month, and religious practice. Consistent with our results, Bulloch et al also reported a lower risk of depression among single, widowed, or divorced older adults compared to those who were married.^[47]^ This association may be partly explained by the caregiver strain experienced by spouses, especially when accompanied by a lack of appreciation.^[48]^ Therefore, supportive interventions for spousal caregivers should aim to alleviate role-related burden and promote mutual gratitude. Older adults living alone face an elevated risk of mental health decline,^[49,50]^ which may be mediated by challenges in self-care and consequent feelings of helplessness and anxiety. To counter this, promoting social activity and bolstering support systems: as recommended by Yoshida et al.^[51]^: are key intervention targets. Nursing home residents are at elevated risk for mental health disorders. Davison et al observed persistently high levels of depressive and anxiety symptoms among residents across multiple facilities in Melbourne,^[52]^ a pattern compounded by social isolation, limited autonomy, and reduced contact with family members.^[53]^ Jun et al further reported that approximately one-quarter of nursing home residents meet criteria for an anxiety disorder, half of whom also experience comorbid depression. These issues may be exacerbated by infrequent contact with relatives or friends and chronic understaffing, which collectively intensify residents’ psychological distress. In this context, structured activity scheduling has shown promise in improving mental health outcomes in nursing home settings.^[54,55]^ For example, group music-rhythm programs enhance cognition, reduce depression/anxiety, and improve life satisfaction.^[54]^ Household income correlates with anxiety and depressive symptoms in older adults.^[43,56]^ Shiba et al demonstrated that retirement exacerbates depressive symptoms in older adults, particularly in men from lower occupational backgrounds.^[43]^ Pan et al demonstrate that China’s New Rural Social Pension alleviates depression in rural elders, especially vulnerable subgroups (women, central residents, low-income households).^[57]^ Therefore, social workers should: advocate for greater investment in rural eldercare services; enhance social security systems to ensure stable pension and healthcare coverage for rural seniors; and facilitate internet access and provide digital literacy training to vulnerable older adults, thereby supporting their financial and psychological preparedness and mental well-being.^[58]^ Contrasting Tan et al finding of religion’s positive mental health effects (varying by race), our study suggests Confucian familial obligations in China may induce role adequacy pressure, increasing anxiety/depression risks.^[59]^ Older adults in economically underdeveloped regions often have outdated ideas and misunderstand religious teachings. This can lead to negative psychological emotions. Establishing elderly universities to deliver accurate religious education can help elders comprehend doctrines, dispel misconceptions, and alleviate afterlife concerns. Organizing faith-based activities (e.g., book clubs, lectures, group events) fosters elderly social inclusion and life enrichment. Notably, this study did not assess spirituality/religiousness as potential mediators between risk factors and depressive and anxiety symptoms. Emerging evidence indicates these factors may buffer or exacerbate psychological outcomes in older adults. For example, religious practices in Greek Orthodox populations reduced anxiety/depression,^[60]^ while COVID-related restrictions impaired life satisfaction.^[61,62]^ In Guizhou’s cultural context (e.g., Buddhism/Taoism), similar mediation may occur through social support or coping mechanisms. Future studies should incorporate validated scales (e.g., Daily Spiritual Experience Scale) to clarify these pathways, especially in high-risk subgroups.

### 4.3. The impact of physical health and family relationship factors on depressive and anxiety symptoms in older adults

Older adults with physical impairments (e.g., back pain, sensory loss), ADL dependence, multiple chronic conditions, family mental health history, or recent stressful events face elevated depressive and anxiety symptoms. These factors reduce self-efficacy and social engagement, which heightens feelings of helplessness and increases vulnerability to anxiety and depression.^[63,64]^ Beyond these psychosocial pathways, a potential biological mechanism may involve chronic inflammation. Many chronic diseases and physical disabilities are associated with a state of elevated systemic inflammation, characterized by increased pro-inflammatory cytokines (e.g., IL-6, TNF-α). This inflammatory state can communicate with the brain, leading to neuroinflammation, alterations in neurotransmitter metabolism (e.g., serotonin and dopamine), and ultimately, the development of depressive and anxiety symptoms. Thus, the link we observed may be partly mediated by a shared underlying inflammatory pathophysiology. To address these challenges, we propose that the provision of psychological support is imperative. Establishing support groups for older persons is recommended to promote communication and mutual support among group members. It is imperative to furnish older adults who have changed within their families with the requisite psychological counseling and livelihood assistance. Regular medical checkups are also paramount. These checkups should include developing individualized chronic disease management plans, including diet, exercise, and medication management. Enhanced socialization: It is recommended that volunteers be recruited to visit the elderly regularly, focusing on providing companionship and support, especially for those who have lost their spouses or have no children to care for them. Safety monitoring: The installation of safety monitoring equipment is crucial to ensure the safety of the elderly within their homes and minimize accidents. Family education: Conduct educational programs for family members of elderly individuals with a history of mental illness to promote understanding of the elderly’s needs and facilitate more effective support. Notably, elderly individuals who travel more than an hour to reach the nearest hospital for medical treatment experience a protective effect against symptoms of depression and anxiety. This may be because elderly individuals who seek long-term medical care tend to pay more attention to their health, which helps them better control chronic diseases and improve their physical condition. This proactive attitude indirectly improves their mental health. For instance, they regularly take their medication and strictly follow their doctor’s instructions for managing chronic diseases.

Consistent with prior research,^[65,66]^ spousal, familial, and community networks are vital for the mental well-being of older adults. These ties enhance psychological well-being and life satisfaction by providing multidimensional support, including emotional, informational, and practical assistance. The protective effect of strong social ties can be further understood through the biopsychosocial model. Psychologically, perceived social support fulfills the fundamental human need for belonging and provides a buffer against the detrimental effects of stress. Biologically, positive social interactions are thought to modulate the body’s stress response systems, for instance, by reducing cortisol production and blunting cardiovascular reactivity to stressors. This downregulation of the stress response may protect against the neuronal damage and dysregulation of the HPA axis that is often observed in mood and anxiety disorders. Social workers should therefore actively maintain elders’ close ties with spouses, children, and key family members by facilitating regular communication to strengthen perceived family support networks.

### 4.4. The impact of lifestyle factors on depression and anxiety symptoms in older adults

Smoking and alcohol consumption have been linked to anxiety and depression in the elderly in early studies.^[67,68]^ We found that sustained heavy smoking (≥20 cigarettes/day over 10–20 years) has profoundly detrimental effects on older adults’ mental health. Long-term smoking is associated with atherosclerosis, which impairs blood flow. Atherosclerosis reduces cerebral blood flow, increasing the risk of cognitive impairment and depression.^[69]^ It is recommended that older adults gradually reduce their smoking and ultimately cease smoking in order to enhance their mental health and quality of life. Additionally, abstaining from alcohol (including spirits and beer) is beneficial for alleviating symptoms of depression and anxiety. Alcohol intake and drinking patterns are closely related to the onset and development of chronic diseases. Long-term drinking in this population often leads to alcohol use disorders, which in turn cause physical and mental health problems, including liver disease, pancreatitis, dementia, and various cancers.^[70]^ Consequently, older adults who consume large amounts of alcohol long-term should consider abstinence. If quitting is difficult, seeking professional support to overcome abstinence-related barriers is advised. Moutai wine, with its rich cultural heritage in Guizhou Province, offers older adults an alternative to alcohol consumption through cultural activities like winery tours and lectures, deepening their appreciation of this tradition. Older adults who regularly consume dairy products, soy products, meat, vegetables, or fruit are less likely to experience symptoms of depression and anxiety. Research indicates that high dairy consumption is associated with improved overall cognitive function, visuospatial memory, and executive functioning.^[71,72]^ The mechanisms through which diet influences mental health are multifaceted. A balanced diet provides essential micronutrients (e.g., B vitamins, zinc, magnesium) that act as cofactors in the synthesis of key neurotransmitters like serotonin and dopamine. Soy products provide high-quality plant protein essential for nerve cell repair and regeneration,^[73]^ and regular intake is linked to reduced depression risk.^[74]^ Meat is a key source of B vitamins, vital for neurotransmitter synthesis and metabolism,^[75]^ which help alleviate anxiety and depressive symptoms.^[76]^ Similarly, regular fruit and vegetable consumption supports cognitive and mental health, as they supply B vitamins (e.g., folate, B6, B12) crucial for nervous system function, neurotransmitter activity, mood regulation, and cognition.^[77,78]^ Therefore, it is recommended that older adults consume sufficient dairy products, soy products, meat, vegetables, and fruits every week. In this study, dietary patterns among older adults had an impact on symptoms of depression and anxiety. Regardless of whether they consumed 2 meals daily (breakfast and dinner), a regular 3-meal diet, or a 4-meal diet including late-night snacks, suboptimal mental well-being was frequently observed. Notably, adherence to a strict 3-meal pattern was associated with the most concerning mental health outcomes. A fixed 3-meal schedule may induce monotony and boredom due to its rigid routine, leading to psychological fatigue and anxiety. A 4-meal pattern often promotes irregular eating and potential overeating, adversely affecting both physical and psychological health. Furthermore, late-night snacking can disrupt sleep quality, further impairing mental health. Skipping dinner results in prolonged overnight fasting and lowered glucose levels, causing hunger, dizziness, and fatigue. These discomforts impair sleep quality and can trigger psychological issues like irritability and anxiety. Therefore, it is recommended that older adults eat small meals more frequently. Incorporating healthy snacks, including nuts, yogurt, and fruit, in moderation between meals can assist in maintaining energy levels and preventing fluctuations in mood.

Moderate sleep duration, defined as 7 to 9 hours, has been identified as a protective factor for mental health in older adults.^[79]^ Moderate participation in recreational activities significantly prevents anxiety and depressive symptoms in older adults. Activities such as chess, poker, pet interaction, square dancing, and tai chi facilitate social engagement, reducing loneliness and enhancing social support. Notably, Guizhou Province possesses a distinct mahjong culture, particularly exemplified by its unique “Catch Chicken Mahjong.” However, contrary to previous research,^[80]^ this study found no significant impact of mahjong activities on older adults’ depressive and anxiety symptoms. This discrepancy may be attributable to mahjong’s association with gambling, which can negatively affect psychological well-being, or to confounding variables inherent in this observational study design (lacking a randomized controlled trial). Based on our findings, we recommend the following activity patterns for older adults: Chess/poker: 1 to 3 days/week (≥2 hours/session); Pet interaction: 4 to 7 days/week (≤15 minutes/session); Square dancing: 4 to 7 days/week (≥30 minutes/session); Tai chi: 1 to 3 days/week (≤30 minutes/session). Hou et al^[81]^ demonstrated a robust positive correlation between physical activity and mental health in older adults. Our findings identified brisk walking and appropriate high-intensity physical activity as protective factors. The benefits of physical activity like brisk walking and Tai Chi may be mediated by increased production of brain-derived neurotrophic factor, which supports neuroplasticity and neuronal survival, and by the regulation of the endocannabinoid system, which contributes to mood stabilization and the “runner’s high” phenomenon. These activities significantly improve physical functions such as cardiorespiratory fitness, muscle strength, and joint flexibility.^[82]^ These improvements reduce chronic disease risk, thereby enhancing overall quality of life and positively impacting mental health. Based on the study findings, individuals should engage in brisk walking 4 to 7 days per week or 15 minutes of daily high-intensity activity (e.g., running, swimming), while considering their physical capabilities. Public health campaigns that promote regular physical activity, balanced nutrition and good sleep hygiene could be an accessible and non-stigmatizing way to improve the mental well-being of an aging population. Reducing domestic responsibilities may preserve elderly mental well-being by facilitating adaptation to age-related social and familial role transitions. This allows energy to be reallocated toward more meaningful activities, such as community participation and family culture transmission: fostering psychological satisfaction and social identity. Our results are confirmed by Zhang et al,^[83]^ who also reported a positive association between internet use and mental health in older adults. Specifically, older adults watching television for >60 minutes daily or using cell phones for ≤30 minutes daily showed significantly improved depressive and anxiety symptoms. This moderate screen time provides an optimal balance to avoid fatigue while maintaining social and cognitive engagement. Interestingly, limited mobile phone use (<1 day per week) also mitigates depressive and anxiety symptoms in older adults. This protective effect may stem from reduced information overload, avoidance of addiction, increased real-world social interaction, engagement in other beneficial activities, and minimized exposure to negative content. Therefore, we recommend that older adults prioritize television as their primary source of information and watch television for at least 4 days per week (at least 1 hour per day).

### 4.5. Implications for public health and stratified management

The identification of 4 distinct subgroups of depressive and anxiety symptoms provides a robust empirical basis for moving beyond a “one-size-fits-all” approach to mental health care for the elderly. Our findings advocate for the implementation of stratified management strategies, where resources and interventions are prioritized and tailored based on an individual’s risk profile and subgroup characteristics.

Based on our results, we propose the following framework for public health action: For the “Severe depressive and anxiety symptoms group” (Class 1): This group requires immediate and intensive intervention. Management should be led by primary healthcare centers in coordination with family members. Key strategies include: Priority Referral: Expedited referral to specialized mental health services for comprehensive assessment and potential pharmacological treatment. Integrated Home Care: Regular home visits by community health workers to provide medication adherence support, basic psychological first aid, and monitor physical health conditions. Family-Centered Support: Provide intensive family education and counseling to improve the home environment and equip caregivers with necessary skills. Social Welfare Linkage: Actively connect these individuals and their families with social security services to address underlying economic hardships. For the “Moderate depressive and anxiety symptoms group” (Class 2): This group would benefit from targeted prevention and structured support within the community. Group-Based Psychotherapy: Offer structured group interventions (e.g., problem-solving therapy, mindfulness-based stress reduction) at community centers to improve coping skills and reduce isolation. Chronic Disease Management Clubs: Integrate mental health screening and support into existing chronic disease management programs. Social Prescription: Community workers can “prescribe” participation in specific social or recreational activities (e.g., calligraphy classes, choir) to build social networks. Respite Care for Caregivers: Provide support services for spousal caregivers to alleviate their burden and improve the quality of care. For the “Mild depressive and anxiety symptoms group” (Class 3): The focus for this large subgroup should be on resilience-building and mental health promotion to prevent symptom escalation. Public Health Education: Launch community-wide campaigns focusing on the importance of physical activity, balanced nutrition, sleep hygiene, and maintaining social connections. Peer-Led Initiatives: Train and support healthier older adults to lead exercise groups (e.g., Tai Chi, square dancing) or peer-support conversations. Digital Health Tools: Develop and disseminate simple, evidence-based mental health information via smartphones or television, which were identified as protective factors. For the “Group without depressive or anxiety symptoms” (Class 4): This group represents an ideal target for universal prevention and the promotion of positive aging. Resource and Advocacy Role: Engage these individuals as volunteers and mental health advocates within their communities to help reduce stigma. Lifelong Learning: Encourage participation in elderly universities to maintain cognitive function and social engagement. Routine Screening: Include brief mental health assessments as part of annual physical checkups to ensure early detection of any emerging issues.

Implementing this stratified framework requires policy support to strengthen the urban-rural mental health service network, train primary care personnel in geriatric mental health, and integrate mental health into existing elderly care and social welfare systems.

## 5. Limitations and outlook

Our study has significant strengths. First, using the statistical method of LCA, we conducted a cross-sectional analysis of the mental health status of older adults aged 65 years and above. We found that the mental health characteristics of this population showed a clustering phenomenon. Secondly, this study analyzed factors influencing depression and anxiety symptoms in older adults across 3 dimensions, incorporating the cultural and regional characteristics of economically underdeveloped areas. The identified risk factors serve as key indicators for preventing these symptoms, enabling more targeted screening, early detection, and focus on at-risk groups. This provides direction and a theoretical foundation for future research. Finally, this study explored risk and protective factors associated with demographic characteristics, physical status, family relationships, and lifestyle, providing individualized interventions for older adults.

However, there are several limitations to this study. First, a methodological limitation of our study pertains to the use of dichotomized variables for the LCA. The original PHQ-9 and GAD-7 items are rated on a 4-point Likert scale, capturing gradients of symptom frequency (e.g., “several days” vs “more than half the days”). By recoding these into binary categories (absent vs present), we inevitably lost some of this nuanced information regarding severity. This process might have simplified the symptom patterns, potentially leading to less differentiated subgroups than if the full spectrum of responses had been utilized. While this approach is common in LCA and can aid in model parsimony and stability, future research would benefit from employing latent profile analysis. Latent profile analysis is specifically designed for continuous indicators and would allow for the identification of subgroups based on the full severity scores of the PHQ-9 and GAD-7 items. This could uncover more refined, severity-based phenotypes of co-occurring depressive and anxiety symptoms in the elderly, potentially enhancing the personalization of intervention strategies. Second, this study used unvalidated instruments to assess current physical condition, family relationships, and living situation, which relied on self-report and thus may have affected the accuracy of the results. When interpreting research findings, keep in mind the limitations of self-reporting methods, particularly when the information is from the past or could be stigmatizing. Additionally, depressive and anxiety symptoms were evaluated using the Chinese versions of the PHQ-9 and GAD-7, respectively. These scales are widely used as self-management tools for assessing depression and anxiety symptoms worldwide.^[84]^ However, these 2 scales were not developed as research tools to assess depressive and anxiety symptoms in the elderly. Future studies should use the 15-item Geriatric Depression Scale (GDS-15)^[4]^ and the Geriatric Anxiety Inventory (GAI-SF)^[5]^ for this purpose. Third, our sample was drawn exclusively from Guizhou Province, a region characterized by a rapidly aging population, significant rural poverty, a high proportion of ethnic minorities, and an uneven distribution of healthcare resources. The unique socioeconomic, cultural, and healthcare access conditions in Guizhou have likely shaped the specific patterns of depressive and anxiety symptoms and their associated factors that we identified. Consequently, the direct application of our latent class structure and the specific risk/protective factor profiles to elderly populations in other provinces of China, or in other countries with vastly different contexts, should be done with caution. The external validity of our findings needs to be established through future research. Fourth, this was a secondary analysis of a cross-sectional survey study, and no causal conclusions could be drawn, which weakens the dynamic analysis of mental health problems in older adults. Fifth, although we assessed multicollinearity using VIFs and found elevated values for 2 smoking-related variables, this may have influenced the precision of their coefficient estimates. Future studies should consider alternative modeling strategies, such as combining correlated variables or using regularization methods, to mitigate this issue. Simultaneously, future studies with independent validation cohorts are recommended to externally validate the stability and generalizability of the risk and protective factors identified in this study. Finally, mental health problems in older adults include not only depression and anxiety but also other mental health problems (e.g., happiness and anger) not included in our study, which may lead to an underestimation of mental health problems in this population.

## 6. Conclusion

In summary, this study provides the first LCA of depressive and anxiety symptoms among individuals aged 65 and older in the specific context of Guizhou Province, China: a resource-limited, multi-ethnic region with distinctive aging challenges. We have demonstrated significant mental health heterogeneity within this population and identified context-specific risk and protective factors. While our findings offer a valuable evidence base for designing stratified mental health interventions in Guizhou and other socioeconomically similar regions, their generalizability to more developed or culturally distinct areas is uncertain. Therefore, we strongly recommend that future research validates the latent class structure and tests the applicability of our proposed stratified management framework through multi-center studies encompassing diverse geographic, economic, and cultural settings across China and beyond.

## Acknowledgments

We extend our sincere gratitude to all participants and staff members who contributed to the initial investigation conducted by the GZCDC.

## Author contributions

**Conceptualization:** Jing Zhang, Feng Li, YunTong Yao, YuQing Huang, YiXia Zhang, YangWen Yu.

**Data curation:** Jing Zhang, Feng Li, YunTong Yao, YuQing Huang, YiXia Zhang, YangWen Yu.

**Investigation:** Jing Zhang, Feng Li, YunTong Yao, YuQing Huang, YiXia Zhang, YangWen Yu.

**Project administration:** Jing Zhang, YunTong Yao, YuQing Huang, YiXia Zhang, YangWen Yu, Xu Luo.

**Resources:** Jing Zhang, Feng Li, YunTong Yao, YuQing Huang, YiXia Zhang, YangWen Yu.

**Software:** Jing Zhang, Feng Li.

**Supervision:** Jing Zhang, YunTong Yao, YuQing Huang, YiXia Zhang, YangWen Yu, Xu Luo.

**Formal analysis:** Feng Li.

**Validation:** Feng Li, Xu Luo.

**Visualization:** Feng Li.

**Funding acquisition:** YangWen Yu, Xu Luo.

**Methodology:** YangWen Yu, Xu Luo.

**Writing – original draft:** Jing Zhang, Feng Li, YangWen Yu, Xu Luo.

**Writing – review & editing:** Jing Zhang, Feng Li, YangWen Yu, Xu Luo.










