# Supplementary material for: A cross-sectional latent class analysis of depressive and anxiety symptoms in people aged 65 and older: Identifying subgroups and related factors
Source: Medicine (Baltimore). 2026 Aug 21;105(34):e50328. doi: 10.1097/MD.0000000000050328 (PMC13502833; doi:10.1097/MD.0000000000050328)
Supplement: Supplementary file 1 [file medi-105-e50328-s001.pdf]

Supplementary Material 1

1 PHQ-9 Scale

In the last year, have you ever had any of the following illnesses that lasted for 2 weeks? If there was more than one episode, please choose the one with the worst symptoms and select the appropriate option for the 2 weeks when the symptoms were worst.

Title (during the fortnight when symptoms are worst)	Not at all.	Several days	More than half the days	Almost daily
1.1 Lack of motivation or interest in work	0	1	2	3
1.2 Feeling low, depressed or hopeless	0	1	2	3
1.3 Difficulty falling asleep, restless sleep or sleeping too much	0	1	2	3
1.4 Tiredness or lack of energy	0	1	2	3
1.5 Loss of appetite or eating too much	0	1	2	3
1.6 Feeling bad about yourself, or feeling like a failure, or letting yourself or your family down	0	1	2	3
1.7 Trouble concentrating on things, such as reading the newspaper or watching TV	0	1	2	3
1.8 Working or talking so slowly that others have noticed or, on the contrary, being irritable or fidgety, moving around more than usual	0	1	2	3
1.9 Thoughts of dying or hurting yourself in some way.	0	1	2	3

2 GAD-7 scale

In the last year, have you ever had any of the following illnesses that lasted for 2 weeks? If there was more than one episode, please choose the one with the worst symptoms and select the appropriate option for the 2 weeks when the symptoms were worst.

Title (during the fortnight when symptoms are worst)	Not at all.	Several days	More than a week	Almost daily
2.1 Feeling nervous, anxious or eager	0	1	2	3
2.2 Inability to stop or control worries	0	1	2	3
2.3 Worrying too much about different things	0	1	2	3
2.4 It's hard to relax.	0	1	2	3
2.5 Unable to meditate due to anxiety	0	1	2	3
2.6 Becoming easily annoyed or impatient	0	1	2	3
2.7 Fear as if something terrible is going to happen	0	1	2	3

3 AD8 scale

Please select whether the older adult has experienced any of the following types of changes in ability due to thinking and memory problems in the last few years.

Title	Changed	Unchanged	Not sure
3.1 Poor judgement (e.g. gullibility, falling for traps or scams, poor financial decisions, inappropriate gifts, etc.)			
3.2 Decrease in interest in hobbies and activities			
3.3 Repeating the same things over and over (e.g. asking the same questions, doing the same things, saying the same things)			
3.4 Difficulties in learning how to use tools, electrical appliances or gadgets (e.g. TV, washing machine, air conditioner, gas cooker, water heater, microwave, remote control, etc.)			
3.5 Forget the correct month and year			
3.6 Difficulty dealing with complex financial issues (e.g. balancing income and expenses, accessing money, paying utility bills, etc.)			
3.7 Difficulty in remembering the appointment time			
3.8 Problems with thinking or remembering in everyday life			
