# Supplementary material for: A cross-sectional latent class analysis of depressive and anxiety symptoms in people aged 65 and older: Identifying subgroups and related factors
Source: Medicine (Baltimore). 2026 Aug 21;105(34):e50328. doi: 10.1097/MD.0000000000050328 (PMC13502833; doi:10.1097/MD.0000000000050328)
Supplement: Supplementary file 2 [file medi-105-e50328-s002.pdf]

# Supplement Material 2

## 1 Variables related to demographic characteristics (n=19)

| Variable | Unit (of measure) | Define variable | Categorical variable | Encoding |
|---|---|---|---|---|
| Age | Year | None | 65-79 | 0 |
| | | | ≥80 | 1 |
| Gender | None | None | Male | 0 |
| | | | Female | 1 |
| BMI | Kg/m² | Waste away | <18.5 | 0 |
| | | Normalcy | 18.5-25 | 1 |
| | | Overweight | 25-30 | 2 |
| | | Obese | ≥30 | 3 |
| Ethnic group | None | None | Han Chinese | 0 |
| | | | Minority | 1 |
| Educational level | None | None | Below elementary school (illiterate) | 0 |
| | | | Elementary education | 1 |
| | | | Secondary education (middle school, high school, junior college) | 2 |
| | | | Higher education (college or university, university and above) | 3 |
| Occupation before retirement | None | None | Unemployed, domestic | 0 |
| | | | Agricultural, forestry, fishery, water conservancy producers | 1 |
| | | | Business, service industry personnel, clerical staff, related personnel | 2 |
| | | | Heads of state organs, party organizations, enterprises, institutions | 3 |
| | | | Professionals,technicians | 4 |
| | | | Operators of production, transportation equipment, related personnel | 5 |
| | | | Other occupations | 6 |
| Marital status | None | None | Married | 0 |
| | | | Other | 1 |

| | | | | |
|---|---|---|---|---|
| | | | (divorced, widowed, unmarried) | |
| Whether living alone | None | None | Yes | 0 |
| | | | Not | 1 |
| Whether living with spouse or partner | None | None | Yes | 0 |
| | | | Not | 1 |
| Whether living with grandchildren | None | None | Yes | 0 |
| | | | Not | 1 |
| Whether living with other relatives | None | None | Yes | 0 |
| | | | Not | 1 |
| Whether living with a nursing home partner | None | None | Yes | 0 |
| | | | Not | 1 |
| Whether living with children | None | None | Yes | 0 |
| | | | Not | 1 |
| Per capita monthly income with spouse | RMB | None | <3000 | 0 |
| | | | 3000-6999 | 1 |
| Religious or not | None | None | Yes | 0 |
| | | | Not | 1 |
| Urban/Rural | None | None | Urban | 0 |
| | | | Rural | 1 |
| Depressive symptoms score | Point | Absence of depression symptoms | 0-5 | 0 |
| | | Presence of depression symptoms | ≥5 | 1 |
| Anxiety symptom score | Point | Absence of anxiety symptoms | 0-5 | 0 |
| | | Presence of anxious symptoms | ≥5 | 1 |
| Dementia score | Point | No cognitive impairment | 0-1 | 0 |
| | | Presence of cognitive impairment | ≥2 | 1 |

## 2 Variables related to physical condition and family relationship (n=13)

| Variable | Unit (of measure) | Define variable | Categorical variable | Encoding |
| --- | --- | --- | --- | --- |
| Physical defects | None | None | Yes | 0 |
| | | | Not | 1 |
| Ability to perform daily activities independently | None | None | Yes | 0 |
| | | | Not | 1 |
| Number of chronic diseases | Type | None | No chronic diseases | 0 |
| | | | 1 chronic disease | 1 |
| | | | ≥2 chronic diseases | 2 |
| Time spent going to nearest hospital | Minute | Short period | <30 | 0 |
| | | Medium time | 30-60 | 1 |
| | | Long time | >60 | 2 |
| Family history of mental illness | None | None | Yes | 0 |
| | | | Not | 1 |
| Family history of Alzheimer's | None | None | Yes | 0 |
| | | | Not | 1 |
| Whether the family had a stressful event in the last year | None | None | Yes | 0 |
| | | | Not | 1 |
| Spousal relationship | Point | Disharmonious relationship | 0-3 | 0 |
| | | Medium term relationship | 4-7 | 1 |
| | | Harmonious relationship | ≥8 | 2 |
| Child relationship | Point | Disharmonious relationship | 0-3 | 0 |
| | | Medium term relationship | 4-7 | 1 |
| | | Harmonious relationship | ≥8 | 2 |
| Son-in-law or daughter-in-law relationship | Point | Disharmonious relationship | 0-3 | 0 |
| | | Medium term relationship | 4-7 | 1 |
| | | Harmonious relationship | ≥8 | 2 |
| Neighboring | Point | Disharmonious | 0-3 | 0 |

| | | | | |
|---|---|---|---|---|
| relation | | relationship | | |
| | | Medium term relationship | 4-7 | 1 |
| | | Harmonious relationship | ≥8 | 2 |
| Number of days per week of contact with relatives and friends | Day | Contactless | 0 | 0 |
| | | Regular contact | 1-3 | 1 |
| | | Frequent contact | 4-7 | 2 |
| Daily contact time with family and friends | Minute | Negative association | 0-30 | 0 |
| | | Active association | ≥30 | 1 |

### 3 Variables related to lifestyle (n=35)

| Variable | Unit (of measure) | Define variable | Categorical variable | Encoding |
|---|---|---|---|---|
| Number of days per week smoking | Day | Never | 0 | 0 |
| | | Occasional | 1-3 | 1 |
| | | Frequent | 4-7 | 2 |
| Number of cigarettes per day | Root | Never | 0 | 0 |
| | | Light | 1-10 | 1 |
| | | Medium | 11-20 | 2 |
| | | Heavy | ＞20 | 3 |
| Year of smoking | Year | Never | 0 | 0 |
| | | Short-term | 1-5 | 1 |
| | | Moderate | 6-10 | 2 |
| | | Long-term | 11-20 | 3 |
| | | Highly addicted | ≥21 | 4 |
| Number of days per week drinking liquor | Day | Never | 0 | 0 |
| | | Occasional | 1-3 | 1 |
| | | Frequent | 4-7 | 2 |
| Amount of white spirit consumed per day | Tael | Never | 0 | 0 |
| | | Light | 1-2 | 1 |
| | | Medium | 2-5 | 2 |
| | | Heavy | ＞5 | 3 |
| History of white wine | Year | Never | 0 | 0 |
| | | Short-term | 1-5 | 1 |
| | | Moderate | 6-10 | 2 |
| | | Long-term | 11-20 | 3 |
| | | Highly addicted | ≥21 | 4 |
| Number of days per week with beer | Day | Never | 0 | 0 |
| | | Occasional | 1-3 | 1 |
| | | Frequent | 4-7 | 2 |
| Beer consumption per day | Tael | Appropriate amount | 0-6 | 3 |
| | | Excessive | ≥6 | 4 |
| Year of beer | Year | Never | 0 | 0 |
| | | Short-term | 1-5 | 1 |
| | | Moderate | 6-10 | 2 |
| | | Long-term | 11-20 | 3 |
| | | Highly addicted | ≥21 | 4 |
| Number of days per week with dairy products | Day | Never | 0 | 0 |
| | | Occasional | 1-3 | 1 |
| | | Frequent | 4-7 | 2 |
| Number of days | Day | Never | 0 | 0 |

| | | | | |
|---|---|---|---|---|
| per week with soy products | | Occasional | 1-3 | 1 |
| | | Frequent | 4-7 | 2 |
| Number of days per week of meat consumption | Day | Never | 0 | 0 |
| | | Occasional | 1-3 | 1 |
| | | Frequent | 4-7 | 2 |
| Number of days of vegetable or fruit consumption per week | Day | Never | 0 | 0 |
| | | Occasional | 1-3 | 1 |
| | | Frequent | 4-7 | 2 |
| Dining pattern | None | None | Breakfast, lunch, dinner | 0 |
| | | | Breakfast, lunch, dinner, evening meal | 1 |
| | | | Breakfast, lunch | 2 |
| | | | Breakfast, dinner | 3 |
| | | | Other | 4 |
| Daily sleeping hours | Hour | Not enough | < 7 | 0 |
| | | Adequate | 7-9 | 1 |
| | | Excessive | > 9 | 2 |
| Number of days per week for mahjong | Day | Never | 0 | 0 |
| | | Occasional | 1-3 | 1 |
| | | Frequent | 4-7 | 2 |
| Mahjong time per day | Hour | Never | 0 | 0 |
| | | Adequate | 0-2 | 1 |
| | | Excessive | >2 | 2 |
| Number of poker or chess days a week | Day | Never | 0 | 0 |
| | | Occasional | 1-3 | 1 |
| | | Frequent | 4-7 | 2 |
| Daily poker or chess time | Hour | Never | 0 | 0 |
| | | Adequate | 0-2 | 1 |
| | | Excessive | >2 | 2 |
| Number of days per week playing with pets | Day | Never | 0 | 0 |
| | | Occasional | 1-3 | 1 |
| | | Frequent | 4-7 | 2 |
| Daily time with pets | Minute | Never | 0 | 0 |
| | | Adequate | 0-15 | 1 |
| | | Excessive | >15 | 2 |
| Number of square dance days per week | Day | Never | 0 | 0 |
| | | Occasional | 1-3 | 1 |
| | | Frequent | 4-7 | 2 |

| | | | | |
|---|---|---|---|---|
| Daily square dance time | Minute | Never | 0 | 0 |
| | | Adequate | 0-30 | 1 |
| | | Excessive | >30 | 2 |
| Number of Tai Chi days per week | Day | Never | 0 | 0 |
| | | Occasional | 1-3 | 1 |
| | | Frequent | 4-7 | 2 |
| Daily Tai Chi Time | Minute | Never | 0 | 0 |
| | | Adequate | 0-30 | 1 |
| | | Excessive | >30 | 2 |
| Number of brisk walking days per week | Day | Never | 0 | 0 |
| | | Occasional | 1-3 | 1 |
| | | Frequent | 4-7 | 2 |
| Daily brisk walking time | Minute | Never | 0 | 0 |
| | | Adequate | 0-30 | 1 |
| | | Excessive | >30 | 2 |
| Number of days of high-intensity physical activity per week | Day | Never | 0 | 0 |
| | | Occasional | 1-3 | 1 |
| | | Frequent | 4-7 | 2 |
| Daily high-intensity physical activity time | Minute | Never | 0 | 0 |
| | | Adequate | 0-15 | 1 |
| | | Excessive | >15 | 2 |
| Number of days of housework per week | Day | Never | 0 | 0 |
| | | Occasional | 1-3 | 1 |
| | | Frequent | 4-7 | 2 |
| Daily time for household activities | Minute | Never | 0 | 0 |
| | | Not enough | 0-30 | 1 |
| | | Adequate | 30-60 | 2 |
| Number of television days per week | Day | Excessive | >60 | 3 |
| | | Never | 0 | 0 |
| | | Occasional | 1-3 | 1 |
| | | Frequent | 4-7 | 2 |
| Daily television time | Minute | Never | 0 | 0 |
| | | Not enough | 0-30 | 1 |
| | | Adequate | 30-60 | 2 |
| | | Excessive | >60 | 3 |
| Number of days per week using cell phones | Day | Never | 0 | 0 |
| | | Occasional | 1-3 | 1 |
| | | Frequent | 4-7 | 2 |
| Time spent using cell phone | Minute | Never | 0 | 0 |
| | | Adequate | 0-30 | 1 |

| | | | | |
|---|---|---|---|---|
| per day | | Excessive | >30 | 2 |
