# Supplementary material for: A cross-sectional latent class analysis of depressive and anxiety symptoms in people aged 65 and older: Identifying subgroups and related factors
Source: Medicine (Baltimore). 2026 Aug 21;105(34):e50328. doi: 10.1097/MD.0000000000050328 (PMC13502833; doi:10.1097/MD.0000000000050328)
Supplement: Supplementary file 3 [file medi-105-e50328-s003.pdf]

Supplementary Material 3

Multiple logistic regression analysis of variables related to demographic characteristics

Variable	Categorical variable	Class 1					Class 2					Class 3				
		B	P-value	OR	95%CI		B	P-value	OR	95%CI		B	P-value	OR	95%CI	
					Lower	Upper				Lower	Upper				Lower	Upper
Age	65-79	0.265	0.086	1.304	0.963	1.764	-0.008	0.917	0.992	0.848	1.159	-0.058	0.277	0.944	0.850	1.048
	≥80 (reference)															
Gender	Male	-0.177	0.244	0.838	0.623	1.128	-0.168	0.034	0.846	0.725	0.987	-0.206	<.001	0.814	0.735	0.901
	Female (reference)															
BMI	<18.5	0.110	0.780	1.116	0.515	2.422	-0.020	0.918	0.981	0.677	1.420	-0.003	0.981	0.997	0.778	1.277
	18.5-25	0.120	0.726	1.127	0.576	2.207	-0.170	0.288	0.843	0.616	1.154	-0.188	0.076	0.829	0.674	1.020
	25-30	-0.208	0.565	0.812	0.400	1.650	-0.400	0.019	0.670	0.480	0.937	-0.202	0.068	0.817	0.658	1.015
	≥30 (reference)															
Ethnic group	Han Chinese	-1.493	<.001	0.225	0.173	0.291	-0.565	<.001	0.569	0.495	0.653	-0.262	<.001	0.770	0.695	0.852
	Minority (reference)															
Educational level	Below elementary school (illiterate)	-0.282	0.757	0.754	0.126	4.502	0.638	0.144	1.893	0.804	4.455	-0.102	0.562	0.903	0.641	1.273

	Elementary education	-0.409	0.653	0.664	0.112	3.946	0.405	0.352	1.500	0.639	3.518	-0.034	0.842	0.966	0.690	1.353
	Secondary education (middle school, high school, junior college)	-0.735	0.427	0.480	0.078	2.936	0.591	0.169	1.806	0.778	4.194	0.051	0.760	1.052	0.761	1.454
	Higher education (college or university, university and above) (reference)															
Occupation before retirement	Unemployed, domestic	0.457	0.006	1.579	1.139	2.188	0.157	0.091	1.170	0.975	1.403	-0.287	<.001	0.750	0.670	0.841
	Agricultural, forestry, fishery, water conservancy producers	-0.027	0.883	0.973	0.677	1.399	0.036	0.685	1.037	0.871	1.233	-0.575	<.001	0.563	0.502	0.631
	Business, service industry personnel, clerical staff, related personnel	-0.047	0.944	0.954	0.252	3.608	-0.374	0.142	0.688	0.417	1.134	0.055	0.583	1.056	0.869	1.285

	Heads of state organs, party organizations, enterprises, institutions	-0.812	0.305	0.444	0.094	2.093	0.070	0.758	1.073	0.685	1.680	-0.623	<.001	0.536	0.414	0.694
	Professionals, technicians	0.143	0.827	1.154	0.321	4.152	-0.133	0.649	0.875	0.493	1.553	0.001	0.994	1.001	0.806	1.243
	Operators of production, transportation equipment, related personnel	-0.368	0.519	0.692	0.227	2.114	0.112	0.595	1.119	0.740	1.691	-0.215	0.082	0.806	0.632	1.028
	Other occupations (reference)															
Marital status	Married	0.063	0.753	1.065	0.720	1.576	0.276	0.005	1.317	1.087	1.596	-0.078	0.270	0.925	0.806	1.062
	Other (divorced, widowed, unmarried) (reference)															
Whether living alone	Yes	0.141	0.521	1.152	0.748	1.773	0.261	0.017	1.299	1.049	1.608	0.107	0.160	1.113	0.958	1.293
	Not (reference)															
Whether living	Yes	0.027	0.890	1.027	0.702	1.502	-0.377	<.001	0.686	0.566	0.832	0.046	0.493	1.048	0.917	1.196

with spouse or partner	Not (reference)															
Whether living with grandchildren	Yes	-0.903	<.001	0.405	0.261	0.629	-0.438	<.001	0.645	0.507	0.821	-0.542	<.001	0.582	0.495	0.683
	Not (reference)															
Whether living with a nursing home partner	Yes	1.653	0.103	5.224	0.715	38.155	1.886	0.003	6.591	1.905	22.801	1.778	0.002	5.916	1.944	18.003
	Not (reference)															
Whether living with children	Yes	-0.472	0.001	0.624	0.469	0.830	-0.451	<.001	0.637	0.549	0.739	-0.081	0.086	0.922	0.841	1.012
	Not (reference)															
Per capita monthly income with spouse	<3000	2.597	0.032	13.424	1.244	144.914	-0.223	0.265	0.800	0.541	1.184	-0.362	<.001	0.696	0.583	0.831
	3000-6999 (reference)															
Religiou	Yes	-0.355	0.458	0.701	0.274	1.792	0.539	0.006	1.714	1.163	2.526	0.177	0.219	1.194	0.900	1.584

s or not	Not (reference)															
Urban/Rural	Urban	0.059	0.696	1.061	0.788	1.430	-0.771	<.001	0.463	0.397	0.539	-0.547	<.001	0.579	0.524	0.640
	Rural (reference)															
Dementia score	0-1	-1.885	<.001	0.152	0.118	0.196	-1.890	<.001	0.151	0.133	0.172	-1.545	<.001	0.213	0.197	0.231
	≥2 (reference)															
Note: The reference category is Class 4.																
