# Supplementary material for: A cross-sectional latent class analysis of depressive and anxiety symptoms in people aged 65 and older: Identifying subgroups and related factors
Source: Medicine (Baltimore). 2026 Aug 21;105(34):e50328. doi: 10.1097/MD.0000000000050328 (PMC13502833; doi:10.1097/MD.0000000000050328)
Supplement: Supplementary file 4 [file medi-105-e50328-s004.pdf]

Supplementary Material 4

Multiple logistic regression analyses of variables related to physical condition and family relationships

Variable	Categorical variable	Class 1					Class 2					Class 3				
		B	P-value	OR	95%CI		B	P-value	OR	95%CI		B	P-value	OR	95%CI	
					Lower	Upper				Lower	Upper				Lower	Upper
Physical defects	Yes	1.726	<.001	5.619	4.133	7.640	1.134	<.001	3.108	2.606	3.706	0.896	<.001	2.450	2.153	2.788
	Not															
Ability to perform daily activities independently	Yes	0.556	0.253	1.744	0.671	4.531	-0.487	0.012	0.614	0.419	0.900	-0.549	<.001	0.577	0.440	0.758
	Not (reference)															
Number of chronic diseases	No chronic diseases	-1.453	<.001	0.234	0.168	0.326	-0.889	<.001	0.411	0.339	0.498	-0.529	<.001	0.589	0.519	0.669
	1 chronic disease	-0.615	<.001	0.541	0.397	0.737	-0.463	<.001	0.629	0.520	0.762	-0.248	<.001	0.780	0.687	0.887
	≥2 chronic diseases															
Time spent going to	<30	0.289	0.391	1.335	0.690	2.585	0.478	0.011	1.613	1.117	2.329	0.545	<.001	1.724	1.335	2.226
	30-60	0.905	0.005	2.472	1.312	4.658	0.611	<.001	1.842	1.282	2.647	0.405	0.002	1.499	1.162	1.934

nearest hospital	>60 (reference)															
Family history of mental illness	Yes	1.112	<.001	3.039	1.616	5.715	0.337	0.123	1.400	0.913	2.148	0.213	0.192	1.238	0.898	1.705
	Not (reference)															
Whether the family had a stressful event in the last year	Yes	1.384	<.001	3.991	1.929	8.258	0.387	0.167	1.473	0.850	2.551	1.039	<.001	2.827	2.004	3.988
	Not (reference)															
Spousal relationship	0-3	-1.427	0.020	0.240	0.072	0.800	0.046	0.863	1.047	0.617	1.777	0.274	0.151	1.315	0.905	1.913
	4-7	-0.037	0.839	0.964	0.678	1.371	0.438	<.001	1.549	1.262	1.902	0.010	0.909	1.010	0.852	1.197
	≥8 (reference)															
Child relationship	0-3	2.191	<.001	8.947	3.530	22.677	0.330	0.280	1.391	0.764	2.533	-0.572	0.028	0.564	0.339	0.939
	4-7	1.842	<.001	6.309	4.708	8.456	0.802	<.001	2.230	1.851	2.687	-0.148	0.041	0.862	0.748	0.994
	≥8 (reference)															
Son-in-law or	0-3	2.303	<.001	10.000	4.188	23.878	1.343	<.001	3.830	2.132	6.881	0.592	0.020	1.807	1.098	2.976

daughter-in-law relationship	4-7	0.153	0.392	1.166	0.821	1.656	0.290	0.009	1.336	1.075	1.660	-0.069	0.400	0.934	0.796	1.095
	≥8 (reference)															
Neighboring relation	0-3	-2.219	0.004	0.109	0.024	0.495	0.175	0.583	1.191	0.638	2.227	0.613	0.011	1.846	1.151	2.960
	4-7	-0.328	0.056	0.721	0.515	1.008	-0.020	0.835	0.980	0.812	1.184	0.726	<.001	2.067	1.853	2.307
	≥8															
Number of days per week of contact with relatives and friends	0	0.935	<.001	2.548	1.783	3.640	1.141	<.001	3.131	2.582	3.796	0.526	<.001	1.693	1.497	1.914
	1-3	-0.097	0.533	0.907	0.669	1.231	0.162	0.032	1.176	1.014	1.364	0.153	<.001	1.165	1.065	1.275
	4-7 (reference)															
Daily contact time with family and friends	0-30	0.051	0.772	1.052	0.746	1.482	-0.812	<.001	0.444	0.383	0.515	0.106	0.054	1.112	0.998	1.239
	≥30 (reference)															
Note: The reference category is Class 4.																
