# Supplementary material for: A cross-sectional latent class analysis of depressive and anxiety symptoms in people aged 65 and older: Identifying subgroups and related factors
Source: Medicine (Baltimore). 2026 Aug 21;105(34):e50328. doi: 10.1097/MD.0000000000050328 (PMC13502833; doi:10.1097/MD.0000000000050328)
Supplement: Supplementary file 5 [file medi-105-e50328-s005.pdf]

Supplementary Material 5

Multiple logistic regression analysis of lifestyle-related variables

Variable	Categorical variable	Class 1					Class 2					Class 3				
		B	P-value	OR	95%CI		B	P-value	OR	95%CI		B	P-value	OR	95%CI	
					Lower	Upper				Lower	Upper				Lower	Upper
Number of days per week smoking	0	1.528	0.018	4.608	1.300	16.329	0.012	0.972	1.012	0.525	1.950	-0.267	0.224	0.766	0.498	1.178
	1-3	1.616	<.001	5.035	2.316	10.948	-0.347	0.248	0.707	0.392	1.273	-0.177	0.316	0.837	0.592	1.185
	4-7 (reference)															
Number of cigarettes per day	0	-3.276	0.016	0.038	0.003	0.546	-0.308	0.732	0.735	0.126	4.284	-1.227	0.004	0.293	0.127	0.678
	1-10	-2.941	0.002	0.053	0.008	0.336	0.130	0.863	1.139	0.260	4.990	-0.807	<.001	0.446	0.278	0.717
	11-20	-2.462	0.012	0.085	0.012	0.583	0.401	0.599	1.493	0.335	6.658	-1.000	<.001	0.368	0.224	0.604
	>20 (reference)															
Year of smoking	0	0.213	0.825	1.237	0.186	8.215	0.659	0.148	1.932	0.792	4.711	0.542	0.098	1.720	0.905	3.268
	1-5	0.213	0.825	1.237	0.186	8.215	0.659	0.148	1.932	0.792	4.711	0.542	0.098	1.720	0.905	3.268
	6-10	0.411	0.735	1.509	0.139	16.387	0.221	0.692	1.247	0.419	3.711	0.716	0.033	2.046	1.058	3.958
	11-20	1.155	0.067	3.173	0.924	10.894	0.485	0.205	1.624	0.767	3.437	-0.029	0.916	0.971	0.564	1.673
	≥21 (reference)															
Number	0	-1.434	0.051	0.238	0.056	1.008	-0.365	0.338	0.694	0.328	1.466	0.367	0.150	1.443	0.876	2.376

of days per week drinking liquor	1-3	-0.121	0.796	0.886	0.355	2.213	-0.180	0.392	0.836	0.554	1.261	0.015	0.908	1.015	0.794	1.297
	4-7 (reference)															
Amount of white spirit consumed per day	0	-0.292	0.849	0.747	0.037	15.096	0.292	0.730	1.339	0.255	7.019	-0.913	0.025	0.401	0.181	0.890
	1-2	-0.066	0.957	0.936	0.084	10.417	1.189	0.135	3.285	0.692	15.603	0.089	0.759	1.093	0.619	1.932
	2-5	0.468	0.718	1.597	0.125	20.358	0.849	0.305	2.337	0.461	11.833	0.205	0.502	1.228	0.674	2.237
	>5 (reference)															
History of white wine	0	2.273	0.032	9.710	1.218	77.442	1.517	0.002	4.557	1.782	11.653	0.478	0.150	1.612	0.842	3.090
	1-5	0.968	0.298	2.633	0.425	16.302	0.821	0.055	2.274	0.984	5.252	-0.293	0.383	0.746	0.386	1.440
	6-10	0.727	0.315	2.069	0.501	8.549	0.264	0.533	1.302	0.567	2.989	-0.165	0.528	0.848	0.508	1.416
	11-20	0.808	0.180	2.242	0.688	7.306	0.557	0.045	1.745	1.013	3.005	-0.290	0.147	0.748	0.506	1.107
	≥21 (reference)															
Number of days per week with beer	0	-0.326	0.720	0.722	0.121	4.297	-0.900	0.033	0.407	0.178	0.928	-0.059	0.858	0.942	0.492	1.806
	1-3	0.826	0.315	2.285	0.456	11.440	0.539	0.222	1.714	0.722	4.070	1.116	<.001	3.052	1.577	5.905
	4-7 (reference)															
Year of beer	0	-1.715	0.042	0.180	0.034	0.941	0.375	0.407	1.455	0.599	3.534	0.132	0.679	1.141	0.612	2.128
	1-5	-5.613	0.001	0.004	0.000	0.114	-1.881	0.012	0.152	0.035	0.658	-2.101	<.001	0.122	0.040	0.378
	6-10	-1.374	0.324	0.253	0.017	3.875	0.073	0.920	1.076	0.257	4.503	-0.072	0.881	0.930	0.362	2.390

	11-20	-0.652	0.486	0.521	0.083	3.264	-0.453	0.503	0.636	0.169	2.393	-0.353	0.456	0.703	0.278	1.777
	≥21 (reference)															
Number of days per week with dairy products	0	-0.706	<.001	0.493	0.343	0.710	0.307	0.002	1.359	1.124	1.644	-0.134	0.021	0.875	0.781	0.980
	1-3	-0.060	0.736	0.942	0.665	1.335	0.193	0.056	1.213	0.995	1.479	-0.028	0.648	0.973	0.863	1.096
	4-7 (reference)															
Number of days per week with soy products	0	-1.042	<.001	0.353	0.190	0.656	-0.842	<.001	0.431	0.324	0.573	0.174	0.028	1.190	1.019	1.389
	1-3	-0.535	<.001	0.585	0.431	0.796	-0.205	0.005	0.814	0.705	0.941	-0.090	0.076	0.914	0.828	1.009
	4-7 (reference)															
Number of days per week of meat consumpti on	0	1.018	0.047	2.767	1.015	7.544	-0.206	0.494	0.814	0.452	1.468	0.405	0.020	1.499	1.065	2.111
	1-3	0.938	<.001	2.556	1.906	3.427	-0.239	0.003	0.787	0.674	0.920	0.141	0.007	1.151	1.038	1.276
	4-7 (reference)															
Number of days of vegetable or fruit consumpti on per week	0	-0.599	0.283	0.549	0.184	1.641	-0.182	0.560	0.833	0.452	1.537	-0.773	<.001	0.462	0.315	0.677
	1-3	-0.167	0.422	0.846	0.562	1.273	-0.500	<.001	0.606	0.464	0.793	-0.587	<.001	0.556	0.459	0.674
	4-7 (reference)															
Dining	Breakfast,	0.653	<.001	1.921	1.405	2.626	-0.340	<.001	0.712	0.614	0.826	0.100	0.058	1.105	0.996	1.226

pattern	lunch, dinner															
	Breakfast, lunch, dinner, evening meal	-0.329	0.669	0.720	0.159	3.256	0.004	0.989	1.004	0.572	1.763	0.377	0.029	1.458	1.040	2.043
	Breakfast, lunch	-0.143	0.841	0.867	0.215	3.500	-0.272	0.425	0.762	0.390	1.487	0.518	0.006	1.678	1.158	2.432
	Breakfast, dinner	-0.692	0.113	0.501	0.213	1.179	-0.072	0.654	0.930	0.678	1.276	-0.173	0.181	0.841	0.653	1.083
	Other (reference)															
Daily sleeping hours	< 7	-0.169	0.783	0.844	0.254	2.809	0.387	0.061	1.472	0.983	2.206	0.135	0.317	1.145	0.878	1.493
	7-9	0.055	0.657	1.056	0.830	1.344	-0.198	0.002	0.820	0.722	0.931	-0.001	0.989	0.999	0.918	1.088
	> 9 (reference)															
Number of days per week for mahjong	0	1.814	0.108	6.133	0.673	55.870	-0.654	0.264	0.520	0.165	1.639	-0.136	0.721	0.873	0.414	1.839
	1-3	0.785	0.237	2.192	0.598	8.042	-0.266	0.392	0.766	0.417	1.409	0.240	0.122	1.272	0.938	1.725
	4-7 (reference)															
Mahjong time per day	0	-1.524	0.132	0.218	0.030	1.584	0.768	0.187	2.156	0.688	6.757	0.181	0.631	1.198	0.573	2.509
	0-2	0.597	0.367	1.817	0.497	6.650	0.001	0.998	1.001	0.523	1.914	-0.284	0.098	0.752	0.537	1.054
	> 2 (reference)															
Number of poker	0	2.770	0.009	15.962	2.014	126.528	0.353	0.538	1.424	0.462	4.386	0.186	0.612	1.204	0.587	2.470

or chess days a week	1-3	0.371	0.595	1.449	0.370	5.676	-0.061	0.846	0.941	0.511	1.733	-0.408	0.033	0.665	0.457	0.968
	4-7 (reference)															
Daily poker or chess time	0	-2.947	0.003	0.053	0.008	0.361	-0.671	0.259	0.511	0.159	1.638	-0.761	0.046	0.467	0.221	0.988
	0-2	-0.276	0.698	0.758	0.187	3.072	0.225	0.540	1.252	0.610	2.567	0.034	0.875	1.034	0.678	1.578
	> 2															
Number of days per week playing with pets	0	1.733	0.185	5.658	0.437	73.342	-0.156	0.788	0.855	0.274	2.668	-0.781	0.070	0.458	0.196	1.067
	1-3	2.143	0.037	8.528	1.135	64.057	0.399	0.393	1.491	0.596	3.731	-0.162	0.664	0.850	0.409	1.769
	4-7 (reference)															
Daily time with pets	0	-1.126	0.320	0.324	0.035	2.981	-0.127	0.828	0.881	0.281	2.760	0.566	0.213	1.761	0.723	4.289
	0-15	-0.744	0.447	0.475	0.070	3.228	0.608	0.202	1.837	0.721	4.677	0.773	0.022	2.166	1.119	4.194
	> 15 (reference)															
Number of square dance days per week	0	-2.729	0.016	0.065	0.007	0.599	-0.598	0.285	0.550	0.184	1.646	-0.897	0.042	0.408	0.172	0.967
	1-3	-1.152	0.035	0.316	0.108	0.922	-1.022	0.001	0.360	0.193	0.670	-1.072	<.001	0.342	0.212	0.552
	4-7 (reference)															
Daily square dance time	0	2.533	0.038	12.591	1.154	137.381	-0.546	0.342	0.579	0.188	1.787	0.558	0.219	1.747	0.717	4.257
	0-30	0.645	0.327	1.906	0.526	6.910	-0.312	0.300	0.732	0.405	1.321	0.081	0.692	1.085	0.726	1.621
	> 30 (reference)															
Number of Tai Chi	0	-0.620	0.636	0.538	0.041	7.029	-0.685	0.237	0.504	0.162	1.569	0.129	0.790	1.137	0.440	2.938
	1-3	-2.022	0.044	0.132	0.018	0.950	-0.167	0.764	0.846	0.286	2.510	-0.585	0.212	0.557	0.222	1.395

days per week	4-7 (reference)															
Daily Tai Chi Time	0	-0.489	0.763	0.614	0.025	14.779	0.647	0.394	1.910	0.431	8.468	-0.565	0.294	0.568	0.198	1.634
	0-30	-0.603	0.636	0.547	0.045	6.680	-0.589	0.406	0.555	0.139	2.223	-1.083	0.011	0.339	0.147	0.778
	> 30 (reference)															
Number of brisk walking days per week	0	0.345	0.638	1.411	0.336	5.923	0.201	0.629	1.222	0.541	2.764	0.378	0.194	1.459	0.825	2.581
	1-3	1.298	0.005	3.662	1.465	9.155	0.234	0.340	1.264	0.782	2.043	0.554	0.001	1.741	1.251	2.422
	4-7 (reference)															
Daily brisk walking time	0	0.323	0.707	1.381	0.257	7.430	-0.690	0.118	0.502	0.211	1.190	-0.032	0.917	0.968	0.524	1.790
	0-30	0.364	0.561	1.439	0.422	4.899	-0.347	0.153	0.707	0.439	1.137	0.265	0.103	1.304	0.948	1.793
	> 30 (reference)															
Number of days of high-intensity physical activity per week	0	-0.052	0.953	0.949	0.165	5.462	-0.620	0.128	0.538	0.242	1.196	-0.468	0.096	0.627	0.361	1.087
	1-3	-0.941	0.233	0.390	0.083	1.832	-0.236	0.515	0.790	0.388	1.608	-0.517	0.055	0.596	0.351	1.012
	4-7 (reference)															
Daily high-intensity physical	0	-0.016	0.985	0.984	0.185	5.222	0.155	0.696	1.168	0.536	2.547	0.099	0.724	1.104	0.636	1.917
	0-15	-1.150	0.234	0.317	0.048	2.105	-1.758	0.001	0.172	0.060	0.497	-1.196	0.001	0.302	0.145	0.631
	> 15 (reference)															

activity time																
Number of days of housework per week	0	0.637	0.067	1.890	0.955	3.739	-0.009	0.964	0.991	0.680	1.446	-0.485	<.001	0.616	0.477	0.796
	1-3	0.133	0.440	1.143	0.814	1.603	-0.048	0.575	0.953	0.806	1.127	-0.193	0.001	0.825	0.734	0.926
	4-7 (reference)															
Daily time for household activities	0	-0.488	0.241	0.614	0.271	1.387	-0.001	0.997	0.999	0.651	1.533	-0.050	0.719	0.951	0.722	1.252
	0-30	-0.217	0.414	0.805	0.478	1.355	-0.009	0.943	0.991	0.769	1.276	-0.048	0.500	0.953	0.828	1.096
	30-60	-0.573	0.048	0.564	0.319	0.996	-0.116	0.397	0.890	0.680	1.165	-0.145	0.057	0.865	0.745	1.004
	>60 (reference)															
Number of television days per week	0	0.198	0.702	1.219	0.442	3.359	0.250	0.395	1.284	0.722	2.285	-0.140	0.498	0.870	0.580	1.303
	1-3	0.137	0.511	1.146	0.763	1.723	0.678	<.001	1.971	1.641	2.367	0.112	0.119	1.118	0.972	1.287
	4-7 (reference)															
Daily television time	0	1.504	0.007	4.499	1.516	13.353	-0.256	0.390	0.774	0.432	1.388	-0.084	0.685	0.919	0.613	1.380
	0-30	1.831	<.001	6.241	3.685	10.569	-0.099	0.326	0.906	0.743	1.103	-0.290	<.001	0.748	0.663	0.845
	30-60	0.787	0.004	2.196	1.278	3.773	-0.128	0.173	0.880	0.732	1.058	-0.185	<.001	0.831	0.747	0.924
	>60 (reference)															
Number of days per week	0	-0.274	0.386	0.760	0.409	1.413	0.027	0.871	1.027	0.743	1.420	-0.253	0.013	0.777	0.636	0.948
	1-3	-0.277	0.231	0.758	0.482	1.193	0.395	<.001	1.484	1.235	1.785	-0.024	0.702	0.976	0.864	1.103
	4-7 (reference)															

using cell phones																
Time spent using cell phone per day	0	-0.359	0.505	0.699	0.243	2.005	0.265	0.434	1.303	0.672	2.526	0.039	0.841	1.039	0.713	1.515
	0-30	-1.492	0.001	0.225	0.090	0.564	0.209	0.496	1.233	0.675	2.251	-0.233	0.172	0.792	0.567	1.107
	> 30 (reference)															
Note: The reference category is Class 4.																
